# Global patterns and edaphic-climatic controls of soil carbon decomposition kinetics predicted from incubation experiments

**DOI:** 10.1038/s41467-023-37900-3

**Published:** 2023-04-15

**Authors:** Daifeng Xiang, Gangsheng Wang, Jing Tian, Wanyu Li

**Affiliations:** 1 State Key Laboratory of Water Resources and Hydropower Engineering Science, Wuhan University, Wuhan, 430072 China; 2 Institute for Water-Carbon Cycles and Carbon Neutrality, School of Water Resources and Hydropower Engineering, Wuhan University, Wuhan, 430072 China

**Keywords:** Carbon cycle, Climate and Earth system modelling, Microbial ecology

## Abstract

Knowledge about global patterns of the decomposition kinetics of distinct soil organic matter (SOM) pools is crucial to robust estimates of land-atmosphere carbon fluxes under climate change. However, the current Earth system models often adopt globally-consistent reference SOM decomposition rates (k_ref_), ignoring effects from edaphic-climate heterogeneity. Here, we compile a comprehensive set of edaphic-climatic and SOM decomposition data from published incubation experiments and employ machine-learning techniques to develop models capable of predicting the expected sizes and k_ref_ of multiple SOM pools (fast, slow, and passive). We show that soil texture dominates the turnover of the fast pools, whereas pH predominantly regulates passive SOM decomposition. This suggests that pH-sensitive bacterial decomposers might have larger effects on stable SOM decomposition than previously believed. Using these predictive models, we provide a 1-km resolution global-scale dataset of the sizes and k_ref_ of these SOM pools, which may improve global biogeochemical model parameterization and predictions.

## Abstract (web summary)

The predictive power of earth system models may be improved by better representation of decomposition processes. Here, the authors use incubation data and machine learning to estimate soil organic matter decomposition kinetic parameters as a reference for global modelling.

## Introduction

Soil contains a variety of soil organic matter (SOM) and stores more than double the carbon (C) in the atmosphere^1^. With intensified global climate change, soil plays an increasingly important role in regulating global C cycling^2^. Earth system models (ESMs) have been conceived to explain global patterns of C stocks and fluxes as well as to project their responses and feedbacks to the climate system^3,4^. Accurate modeling of SOM decomposition processes in ESMs is therefore critical for understanding carbon-climate feedbacks^2,3,5^. The SOM decomposition processes have been modeled to be regulated by biotic and abiotic factors, including microbial activity^6^, soil texture^7^, soil pH^8^, temperature, and moisture conditions^9,10^. Contemporary ESMs typically represent complex SOM decomposition with a few SOM pools via first-order kinetics, where each SOM pool often has a globally consistent reference (or potential) decomposition rate^11,12^. To calculate the actual time-variant decomposition rate, the reference decomposition rate (k_ref_) is further modified by local soil and environmental conditions, such as soil depth, temperature, and moisture, in ecosystem models and ESMs^9,13–17^. However, the k_ref_ of SOM from different locations is generally developed with their own calibration datasets, which is divergent because of diverse edaphic-climatic conditions^18–20^. Thus, challenges remain in quantifying the effects of potential edaphic-climate heterogeneity on the k_ref_ in ESMs, largely owing to the lack of the synthesis of global-scale data, which might introduce bias in global-scale simulations and lead to diverse and unrealistic global C cycle projections^15,21^.

A number of past synthesis studies have been done to examine the sizes of different SOM pools and their respective first-order decomposition rates using laboratory incubation data^22–30^. Acceptable performances have been achieved through fitting incubation datasets by first-order kinetics models with one, two, or three pools^31–33^. The two-pool models are often characterized by a fast pool and a slow pool^34,35^, and a third pool called “passive pool” is commonly added to the three-pool model^36,37^. The fast (or active) SOM is mainly comprised of fresh plant and animal residues that are readily decomposed in a short time, e.g., from a few days to one year^13^. The passive SOM is often physicochemically protected, making it difficult to access and decompose by microorganisms^38^, and its turnover usually lasts for hundreds to thousands of years^13^. The slow SOM, consisting primarily of detritus as well as partially decomposed cells and tissues, often requires decades to decay, which is somewhere between fast and passive SOM^13,39^. The dramatic variation of k_ref_ among different SOM pools is primarily an ecosystem property, including physicochemical and biological impacts of environmental factors^40^. For convenience, these pool names (fast, slow, and passive) will be adopted in this study to distinguish SOM pools with varying k_ref_. In short, these incubation-based model-data integration studies show that k_ref_ varies widely across soils and ecosystems even under the same laboratory conditions (e.g., temperature and moisture). However, far less attention has been paid to the synthesis and generalization of the abundant laboratory-derived first-order kinetics parameters for more accurate representation of heterogeneous SOM decomposition processes in ESMs^41^. It is yet unsure whether certain patterns hold across diverse soils at the global scale.

**Fig. 1:**
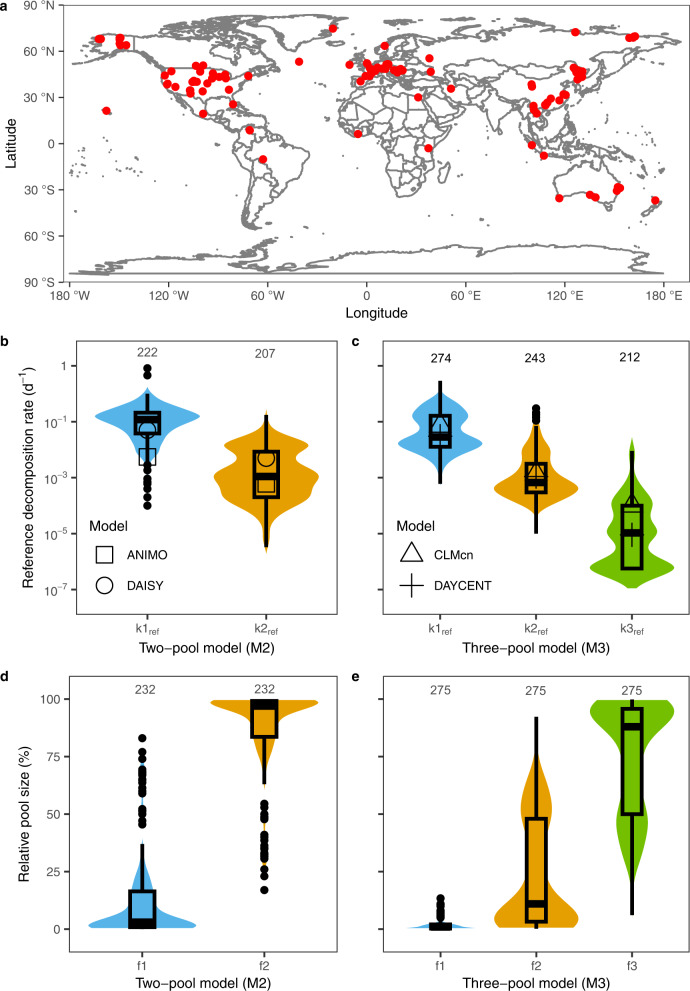
Geographic locations of soil sampling sites and comparison of soil organic matter (SOM) decomposition kinetics parameters. **a** Global distribution of sampling sites. **b**, **c** Reference decomposition rates (k1_ref_, k2_ref_, and k3_ref_) for the fast, slow, and passive SOM pool in the two-pool model (M2) and the three-pool model (M3), respectively. **d**, **e** Relative sizes (f1, f2, and f3) for the fast, slow, and passive SOM pool in the two- and three-pool model, respectively. In **b**–**e** the band reflects the probability density distribution of parameter values, the box represents the first and third quantile, the horizontal line in the box represents the median value, the vertical lines represent minimum and maximum values, the solid dots represent outliers, and the numbers shown in each panel represent sample sizes. The four models are ANIMO^46^, DAISY^47^, CLMcn^13^, and DAYCENT^48^.

Here, based on literature-reported soil incubation experiments and public datasets such as ISCN (the International Soil Carbon Network) and SIDB (the Soil Incubation DataBase)^29,42^, we attempted to fill this gap by generating the global distribution of SOM decomposition kinetics parameters as a reference for global modeling. To address this need, we compiled a dataset with 859 records from 59 laboratory incubation experiments with soils from diverse climate zones and ecosystems (see Fig. 1a and Supplementary Data). We focused on the k_ref_ of different SOM pools, as well as the initial size of each pool (as a percentage of the bulk SOM pool). To explore the relationship between the decomposition kinetics parameters and edaphic-climatic variables^43^, we examined three models, i.e., the traditional multiple linear regression (MLR) and two machine learning approaches—gradient boosting machine (GBM) and random forest (RF) (see Methods). We primarily focused on edaphic-climatic variables as they have generally been used as a proxy to represent the variation in soil community activity mediating global biogeochemical cycling^44,45^. We found that the machine learning methods, especially RF, outcompeted MLR in predicting the decomposition kinetics parameters. Analysis of variable importance based on the RF model shows that soil texture (clay and sand fraction) had the most significant impact on the decomposition of the fast SOM pool, while pH dominated that of the slowest SOM pool. Accordingly, the k_ref_ of different SOM pools exhibited remarkable regional characteristics on a global scale and vary dramatically with latitude.

## Results

### Variability of the first-order decomposition kinetics

Our results revealed a significant difference in the fast-pool reference decomposition rate (k1_ref_) between the two-pool model (M2) and the three-pool model (M3) as per the Kruskal-Wallis (KW) test (*p* < 0.001, Figs. 1b and 1c), but no significant difference in the decay rate of the slow pool (k2_ref_) between these two models (*p* = 0.28; Figs. 1b and 1c). The parameter k1_ref_ had a median of 0.12 d^−1^ (90% Confidence Interval (90% CI): 3.7 × 10^−3^–0.59 d^−1^) in the two-pool model but 0.029 d^−1^ (90% CI: 4.2 × 10^−3^–0.50 d^−1^) in the three-pool model. As for the slow pool, the medians of k2_ref_ were 1.1 × 10^−3^d^−1^ (90% CI: 5.02 × 10^−5^–2.00 × 10^−2^ d^−1^) and 6.8 × 10^−4^d^−1^ (90% CI: 1.21 × 10^−4^–3.80 × 10^−2^ d^−1^) for the two- and three-pool model, respectively. The reference decomposition rate of the passive pool in the three-pool model was 1−3 orders of magnitude lower than that of the slow pool (median k3_ref_ = 1.06 × 10^−5^ d^−1^ with 90% CI of 5.30 × 10^−7^–7.00 × 10^−4^ d^−1^).

The k_ref_ values of typical SOM decomposition models are generally within the 50% CI of our synthetic analysis. As a typical two-pool model, the ANIMO model^46^ sets the k_ref_ of fast and slow pool to 5.5 × 10^−3^ d^−1^ and 6.0 × 10^−4^ d^−1^, respectively; while the DAISY model^47^ sets them to 5.0 × 10^−2^ d^−1^ and 5.0 × 10^−3^ d^−1^ (Fig. 1b). In the CLMcn model^13^, the default k_ref_ value of fast, slow and passive pool are 7.1 × 10^−2^ d^−1^, 1.4 × 10^−3^ d^−1^, and 1.0 × 10^−4^ d^−1^, respectively, while these values are 3.0 × 10^−2^ d^−1^, 1.1 × 10^−3^ d^−1^, and 9.0 × 10^−6^ d^−1^, respectively, in the DATCENT model^48^ (Fig. 1c). Except for the fast pool in the two-pool ANIMO model, the k_ref_ values in these four typical models are within the 50% CI of the laboratory incubation experimental data, indicating that our synthesis of lab-derived first-order kinetics were representative. Additionally, the performances of the fitted first-order model in our compiled dataset were satisfactory with almost all R^2^ (coefficient of determination) greater than 0.8 (see Supplementary Fig. 1), further strengthening our confidence in the adequacy of the compiled dataset.

### Predictive modelling of SOM decomposition kinetics parameters

We first attempted to predict the first-order decomposition parameters (k_ref_ and relative sizes) by eleven explanatory variables, including (i) two climatic variables: mean annual precipitation (MAP, mm) and mean annual temperature (MAT, °C), which reflect the effect of regional climate characteristics; (ii) five edaphic variables: sand fraction (Sand, %), clay fraction (Clay, %), soil pH (pH), soil organic carbon content (SOC, g kg^−1^), and microbial biomass carbon content (MBC, g C m^−2^); (iii) two topographic variables: elevation (Elev, m) and terrain slope (Slope, degree or °); (iv) one vegetation variable: normalized difference vegetation index (NDVI, dimensionless); and (v) one variable representing the experimental condition: laboratory incubation temperature (IncT, °C).

The Spearman correlation analysis shows that there was a weak or little correlation between any two of these explanatory variables, except a high correlation between MAP and MAT (Spearman correlation coefficient *ρ* = 0.822) and between Elev and Slope (*ρ* = −0.775) (Supplementary Fig. 2). Feature selection was then adopted to find the best subset of explanatory variables toward efficient modeling^49^. We used the Akaike Information Criterion (AIC)^50^ to select the best model among the models trained with and without feature selection. AIC accounts for both model fitting performance (the mean squared error) and complexity (the number of explanatory variables and the number of observations) and a lower AIC means better performance.

Compared to the models trained with all the eleven explanatory variables, models trained with feature selection achieved a lower AIC in most of the cases pertaining to the three methods (GBM, RF, and MLR) and eight first-order model parameters (Supplementary Fig. 3; Supplementary Table 1): two reference decomposition rates (M2-k1_ref_ and M2-k2_ref_) and one pool size (M2-f1) in the two-pool model (M2), and three reference decomposition rates (M3-k1_ref_, M3-k2_ref_, and M3-k3_ref_) and two pool sizes (M3-f1 and M3-f2) in the three-pool model (M3). We did not need to predict the slow pool size (M2-f2) or the passive pool size (M3-f3) since they could be calculated when M2-f1, M3-f1, and M3-f2 were determined. Therefore, in the following, we mainly focused on the results with respect to the overall best model trained with feature selection.

**Fig. 2:**
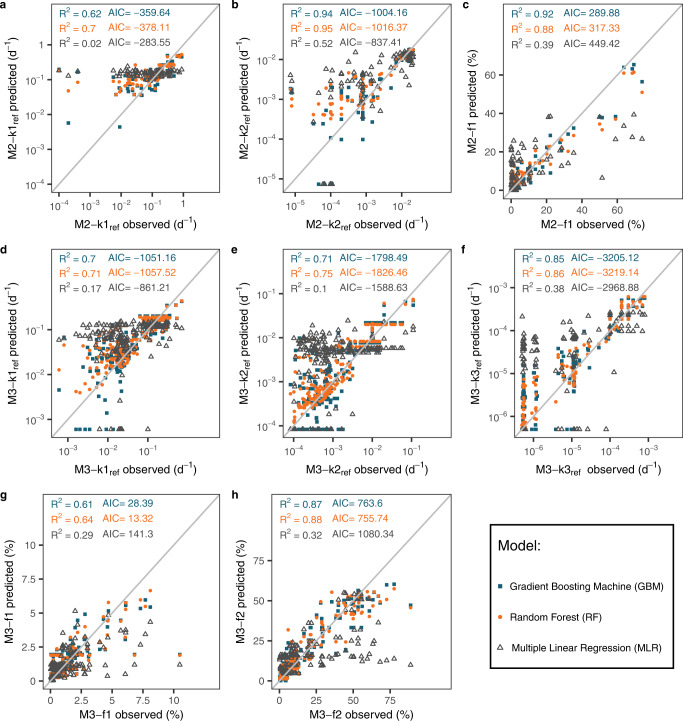
Modeling performance of the Gradient Boosting Machine (GBM), the Random Forest (RF), and the Multiple Linear Regression (MLR) with feature selection. **a**, **b** Reference decomposition rate of the fast (M2-k1_ref_) and slow pool (M2-k2_ref_) in the two-pool model (M2). **c**, Relative size of the fast pool (M2-f1) in the two-pool model. **d**, **e**, **f** Reference decomposition rate of the fast (M3-k1_ref_), slow (M3-k2_ref_) and passive pool (M3-k3_ref_) in the three-pool model (M3). **g**, **h** Relative size of the fast (M3-f1) and slow pool (M3-f2) in the three-pool model. R^2^ denotes the coefficient of determination. AIC denotes the Akaike Information Criterion (see Eq. 11 in Methods).

Our analyses indicate that the RF model, with the lowest AIC and the highest R^2^ (coefficient of determination) and *ρ*_c_ (concordance correlation coefficient^51^, see Methods) was slightly better than the GBM model in predicting the first-order model parameters (reference decomposition rates and SOM pool sizes) (Fig. 2). Results show consistent model performances evaluated by the two metrics (i.e., R^2^ and *ρ*_c_, see Supplementary Table 1). The machine learning models (RF and GBM) performed significantly better than the traditional MLR model, revealing non-linear relationship between the kinetics parameters and the explanatory factors. The R^2^ values of the RF model were 1.83–35 folds higher than those of the MLR method for the two-pool model (Fig. 2a–c) and the three-pool model (Fig. 2d–h). In addition, the RMSEn (normalized root mean square error, see Methods) values of RF were 1.41–3 times lower than those of MLR (Supplementary Table 1). The RF method performed well in both model training (randomly selecting 75% of the full dataset; R^2^ = 0.62–0.94) and model testing (the remaining 25% data; R^2^ = 0.52–0.77) of the eight first-order model parameters except M2-k1_ref_, where a low R^2^ was found in model testing (see Supplementary Table 1). This indicates that RF could be used to reliably predict the decomposition kinetics parameters. The use of the full dataset (i.e., 100% for training) in the RF method slightly improved the performance compared to the model trained with 75% of the dataset (Supplementary Table 1). In view of the overall higher predictive power of RF than the other two approaches (i.e., GBM and MLR), we further used the RF models trained with the full dataset to analyze the relative importance of explanatory variables and predicted the first-order model parameters, particularly the decomposition rates, at the global scale.

### Relative importance of explanatory variables

**Fig. 3:**
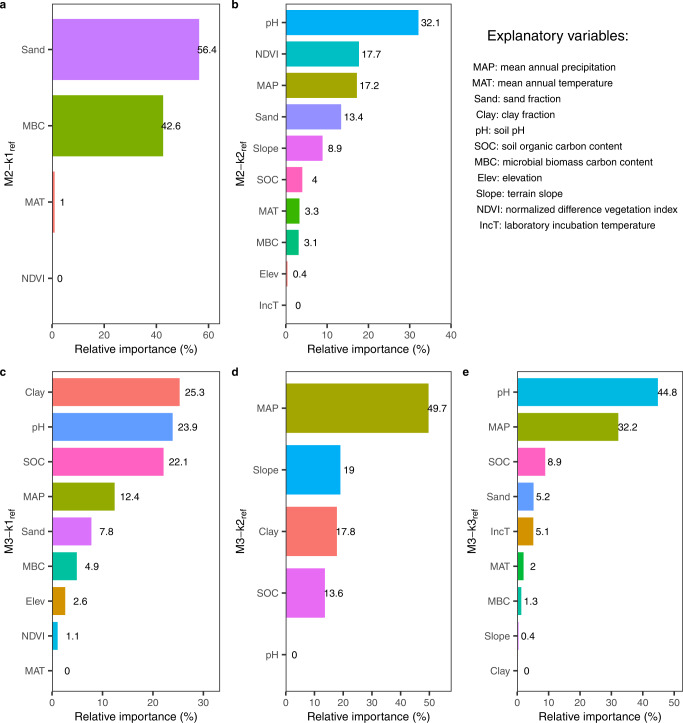
Relative importance of explanatory variables screened out by feature selection in predicting the reference decomposition rates (k_ref_) by the Random Forest (RF) model. **a**, **b** Variable importance of the fast pool (M2-k1_ref_) and slow pool (M2-k2_ref_) in the two-pool model. **c**, **d**, **e** Variable importance of the fast (M3-k1_ref_), slow (M3-k2_ref_) and passive pool (M3-k3_ref_) in the three-pool model. The sum of the relative importance scores of these variables is 100%.

The RF-based variable importance analyses revealed that the dominant predictors for k_ref_ varied across the SOM pools (Fig. 3). In the two-pool model, Sand (56.4% in terms of its relative importance) dominated k_ref_ of the fast pool (M2-k1_ref_), followed by MBC (42.6%) (Fig. 3a). However, soil pH (32.1%), NDVI (17.7%), and MAP (17.2%) became the most important predictors for the slow pool decay rate (M2-k2_ref_) (Fig. 3b).

As for the three-pool model (M3), Clay (25.3%), pH (23.9%), and SOC (22.1%) were the primary regulators for the fast-pool decomposition rate (M3-k1_ref_). As for M3-k2_ref_, the top three influencing variables were MAP (49.7%), Slope (19%), and Clay (17.8%), whereas soil pH (44.8%), MAP (32.2%), and SOC (8.9%) ranked top three among all factors in predicting the passive-pool decay rate (M3-k3_ref_).

In short, soil texture and pH prevailed in the k_ref_ of the fast pool and the slowest pool (slow pool in M2 and passive pool in M3), respectively, whereas MAP stood out in predicting M3-k2_ref_. Terrain slope only had an important impact on M3-k2_ref_. Similar results appeared when all the eleven predictors were considered without feature selection (Supplementary Fig. 4). The partial dependence plots presented high variabilities in the explanatory variables for predicting k_ref_ (Supplementary Fig. 5). More specifically, soil texture, especially sand fraction, had a moderate effect on the two-pool (M2) k_ref_ across the entire value range (Supplementary Fig. 5a and 5b) but a strong effect on the three-pool (M3) k_ref_ at both ends of the value range (Supplementary Fig. 5c and 5e). Climatic factors, particularly MAP, exhibited an intense influence on M3-k2_ref_ and M3-k3_ref_ at both ends (Supplementary Fig. 5d and 5e). However, pH, the dominant controller, showed a subtle marginal effect on k_ref_ (Supplementary Fig. 5c and 5e), which was likely due to variable interactions and vulnerable explanatory power of the partial dependence for complex models^52,53^.

### Global prediction of decomposition kinetics parameters

The three-pool first-order models represent more detailed description of the SOM decomposition kinetics. In addition, the three-pool model presented similar features to the two-pool model as per the variable importance (Fig. 3). Consequently, we predict the first-order kinetics parameters as per the three-pool model (M3) at global scale based on the best RF model with feature selection.

**Fig. 4:**
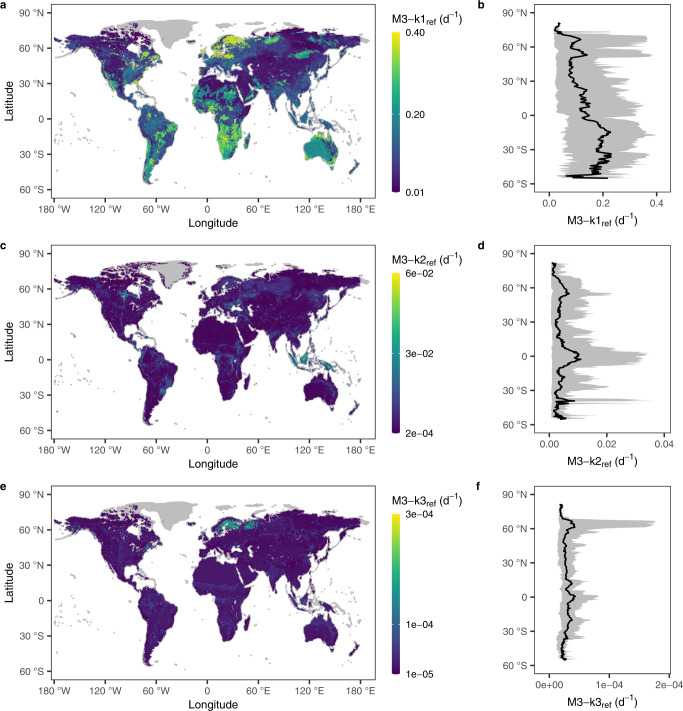
Global distribution and latitudinal pattern of the reference decomposition rates (k_ref_) of the three soil organic matter (SOM) pools predicted by the Random Forest (RF) model with feature selection. **a, b** fast pool (M3-k1_ref_). **c**, **d**, slow pool (M3-k2_ref_). **e**, **f** passive pool (M3-k3_ref_). Data in **b**, **d** and **f** are presented as mean values and 90% confidence intervals at the respective latitude.

For the fast pool (M3-k1_ref_), the predicted values spanned across two orders of magnitude (1.42 × 10^−2^–4.42 × 10^−1^ d^−1^) with a median of 8.57 × 10^−2^ d^−1^ (90% CI: 2.21 × 10^−2^–3.24 × 10^−1^ d^−1^) at the global scale, which exhibited significant spatial variability and varied sharply with latitude (Figs. 4a and 4b). Significantly larger M3-k1_ref_ values (in yellow color) were mainly distributed in coastal areas, such as northwestern Europe, eastern Asia as well as eastern and western part of North America. By contrast, extremely low M3-k1_ref_ values (in dark blue color) were mainly located in highlands represented by the Mongolian Plateau and part of desert areas in Africa (Fig. 4a). Along the latitude, M3-k1_ref_ exhibits significant variability with the most active decomposition activity occurring at around 15 degrees of southern hemisphere and much lower values beyond 30 degrees north latitude (Fig. 4b).

For the slow pool (M3-k2_ref_), SOM tended to decompose faster in regions of northern North America, southeastern South America and southern Africa, with M3-k2_ref_ ranging from 2.08 × 10^−4^ to 5.76 × 10^−2^ d^−1^ (median = 1.99 × 10^−3^ d^−1^ and 90% CI: 6.61 × 10^−4^–1.36 × 10^−2^ d^−1^) (Fig. 4c). Furthermore, M3-k2_ref_ manifested a bimodal pattern and reached peaks around the equator and c.a. 60 degrees north latitude (Fig. 4d). The k_ref_ of the passive pool (M3-k3_ref_) was about two orders of magnitude lower than M3-k2_ref_ and ranged between 9.60 × 10^−6^ and 2.53 × 10^−4^ d^−1^ (median = 2.57 × 10^−5^ d^−1^ and 90% CI: 1.58 × 10^−5^–5.54 × 10^−5^ d^−1^). Contrary to the slow pool, M3-k3_ref_ was higher in desert areas such as the Sahara Desert but lower in the Amazon Rainforest (Fig. 4e). Similar to the fast and slow pools, M3-k3_ref_ fluctuated greatly with latitude and attains higher values around the equator and c.a. 60 degrees north latitude, with extremely low values appearing in areas above 70 degrees north latitude (Fig. 4f).

In addition, we predicted the relative sizes of SOM pools in the three-pool model. Results indicate that M3-f3 shares the largest proportion of SOM and M3-f1 is the smallest one globally (Supplementary Fig. 6). M3-f1 was expected to averagely share a proportion of 3.0% (median = 2.6%, 90% CI: 0.9%–5.7%). In high latitudes (e.g., greater than 60 degrees) of the northern hemisphere, highlands (represented by the Qinghai-Tibet Plateau) and tropical rainforest areas represented by the Amazon Rainforest, M3-f1 was smaller compared to other regions (Supplementary Fig. 6a and 6b). As for the slow pool, the predicted pool sizes (M3-f2) had an average of 30.1% (median = 31.7%, 90% CI: 5.8%–47.6%) and inclined to be lower in high (e.g., > 60 degrees) northern latitude areas, especially the Arctic, whereas higher proportions were expected to occur in low latitudes, especially the tropical rainforest region in Africa (Supplementary Fig. 6c and 6d). Correspondingly, M3-f3 had a global average of 66.9% (median = 64.6%, 90% CI: 50.3%–93.0%), which is larger than the sum of the other two pool sizes. As opposed to the fast and slow pools, M3-f3 was anticipated to obtain higher values in high northern latitudes (Supplementary Fig. 6e and 6f).

**Fig. 5:**
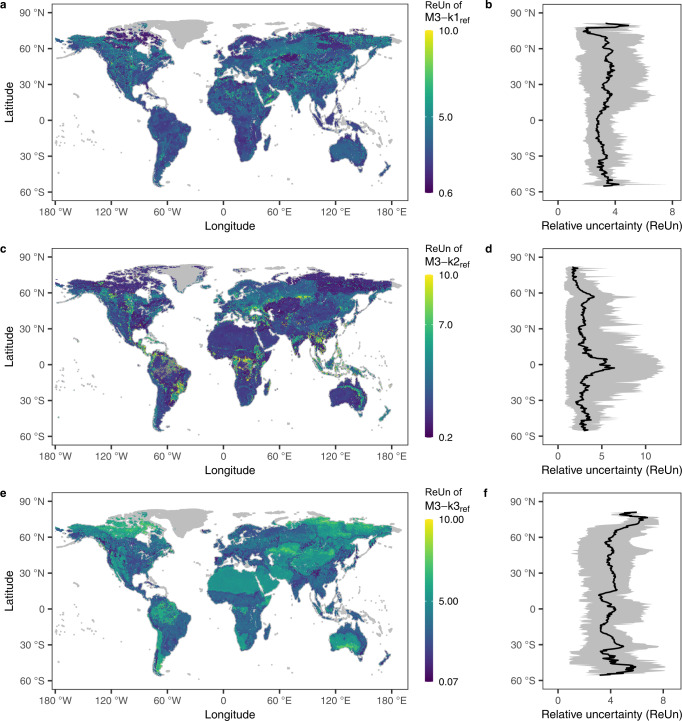
Global relative uncertainty (ReUn) and latitudinal pattern of the reference decomposition rates (k_ref_) of the three soil organic matter (SOM) pools predicted by the Random Forest (RF) model with feature selection. **a**, **b** fast pool (M3-k1_ref_). **c**, **d** slow pool (M3-k2_ref_). **e**, **f** passive pool (M3-k3_ref_). Data in **b**, **d** and **f** are presented as mean values and 90% confidence intervals at the respective latitude.

The uncertainty in the predicted k_ref_ across globe exhibited high spatial variability when considering the uncertainty of input data or the RF model structure. The input global dataset, e.g., soil pH, had relative uncertainty (ReUn = Width_90%CI_/Mean, see Methods) between 0.15 and 0.79, with an average ReUn of 0.49 globally. (Supplementary Fig. 7). The corresponding ReUn of k_ref_ increased with the complexity of the SOM pool, showing global average ReUn of 0.14, 0.44, and 0.84 for M3-k1_ref_, M3-k2_ref_, and M3-k3_ref_, respectively (Supplementary Fig. 8). However, the global mean ReUn of k_ref_ caused by the model structure uncertainty (see Methods) was comparable among the three pools (Fig. 5), but much higher than the ReUn due to the pH uncertainty, especially in regions with poor data, such as the Amazon Rainforest, the Australian desert areas and high latitudes (e.g., >60 degrees north).

## Discussion

### Important factors controlling SOM decomposition rates of different pools

While there is no consensus on the best way to partition bulk SOM into distinct fractions with internally homogeneous characteristics and turnover rates, the consistent differences across fractions in our analysis suggest that it is acceptable to partition bulk soil into conceptual pools that differ in their turnover rates owing to abiotic and biotic mechanisms^38,44^. The relationships of these SOM fractions with climatic-edaphic factors can offer insights into the sensitivity of soil C to climate change and anthropogenic activities.

As a key proxy of soil physical properties, soil texture^54^, especially clay content, exhibited strong influence on M3-k1_ref_ and M3-k2_ref_. Higher soil respiration was generally measured from finer-textured soil with a higher proportion of clay within a specific threshold, where microbial activity was higher as a result of greater soil water holding capacity and nutrient availability^55–57^. However, a higher clay content means a potentially higher proportion of mineral-associated organic matter which could be prevented from microbial utilization, leading to the reduction of SOM decomposition rates^58,59^. Additionally, although water holding capacity was found higher in finer-textured soil^60^, soil moisture under laboratory conditions was usually maintained at a constant value (e.g., 60% water holding capacity), resulting in the inability to assess the direct impact of soil water content on soil respiration^61^. There is possibly a more nuanced indirect effect but that is not explored right now in this study.

The influence of MAP on the reference SOM decomposition rates in the three-pool model ranked first in the slow pool and second in the passive pool. As indicated by previous studies, soil respiration tended to exhibit a negatively asymmetric relationship with precipitation and be more sensitive to decreasing precipitation in arid or semiarid grassland ecosystem such as the Northern Tibet Plateau and Inner Mongolia^62–64^. However, in humid areas such as forests and river deltas, the relationship was positively asymmetric with relatively lower increment in precipitation and negatively asymmetric with relatively higher precipitation^65,66^. The inconsistent soil respiration responses to precipitation between different ecosystems could be explained by the differential responses of plant production and microbial communities^67,68^. Specifically, increasing precipitation in an arid or semiarid area could increase the bacterial and fungal abundance^67^, stimulating the release of soil carbon under laboratory incubation; while a small amount of precipitation in humid area could rapidly result in the saturation of soil moisture and the reduction of oxygen diffusion, depressing biological activities of roots and microorganisms^65,69,70^. In addition, terrain slope has a significant impact on SOC storage by affecting the transport and migration of soil nutrient and soil moisture^71^, thus affecting SOM decomposition rate.

We show that pH is the most important predictor for the reference decomposition rate of the passive pool (M3-k3_ref_). Studies have shown that pH has a direct impact on microbial activities and enzyme production, and either low or high pH could contribute to reduction of soil respiration^72,73^. Soil acidification could constrain agronomic productivity and increase the concentration of toxic metal cations which restrain the growth and maintenance of microorganisms^74,75^, while soil salinization prevents microorganisms from effectively utilizing SOM^76,77^. Soil pH consistently explains a large proportion of the presence of bacterial functioning and the variation in bacterial phyla across studies^44^. It has been suggested that bacteria are more sensitive to pH variation with the highest activation under a neutral pH condition, whereas, fungi have a wider optimal survival pH ranging from 5 to 9 units^74,77,78^. Therefore, a change in pH generally has a more significant impact on bacteria than fungi. This suggest that bacterial decomposers might have larger effects on the decomposition of passive SOM than previously believed^45^, as indicated by recent work that both fungi and bacteria are entailed in breaking down complex substrates in soil^79^. Passive SOM is a mixture of organic substances that have been modified from their original form. While fungi are the dominant decomposers of plant-derived complex compounds (such as lignin and cellulose)^77,80,81^, more bacteria are capable of decomposing fungal and bacterial biomass that exist in the passive SOM pool^79^.

In summary, according to our synthesis of laboratory incubation data, soil texture and pH prevail in the decomposition rates of the fast pools (fast pool in M2 and M3) and the slowest pool (slow pool in M2 and passive pool in M3), respectively, followed by climatic conditions (particularly MAP).

### Regional characteristics of predicted reference SOM decomposition rates

Global-scale prediction of SOM persistence and vulnerabilities under climate change requires the ability to accurately quantify SOM decomposition rates^82^. Our results show that k_ref_ comprises a spectrum ranging from 9.60 × 10^−6^ to 4.42 × 10^−1^ d^−1^. The parameterization of terrestrial C models and ESMs to better predict global C dynamics requires building a better representation of the reference decomposition rates of different SOM pools globally^83^.

The global prediction of kinetic parameters herein depicts strong spatial heterogeneity, which is directly related to the relative importance and distribution characteristics of the edaphic-climatic predictors (see Supplementary Fig. 9). The following discussion is based on our global prediction of SOM kinetics parameters derived from the compiled soil incubation dataset. Noting that the soil samples in this study are under-represented in areas such as the Arctic, the Sahara Desert and the Amazon Rainforest. In tropical rainforest regions of South America (i.e., the Amazon Rainforest) and Africa, the k_ref_ values of the fast and slow pools are higher than adjacent areas, while the opposite was found for the passive pool, where higher k_ref_ values mainly result from relatively lower clay content and pH (Supplementary Fig. 9d and 9f). Precisely, soil in humid tropical forests with slightly lower clay content is less probable to encounter physical protection of mineral-associated organic matter, resulting in higher k_ref_ of the fast and slow pools^58^. However, the significantly lower pH (e.g., <5 units) constrains fungal decomposition of chemically-recalcitrant SOM^84^. In the Australian desert areas, the response of k_ref_ to the explanatory factors is contrary to that of the Amazon Rainforest, which is probably because the relatively high pH (e.g., 7−9 units) limits bacterial activities but maximizes fungal energy efficiency^78,85^. On the Tibetan Plateau, the extremely high altitude tends to be accompanied by longer freezing period restricting diffusion of substrate as well as microbial activities, which makes the impact of elevation on SOM decomposition noticeable and induces all SOM pools especially passive pool to decompose slowly though the soil has similar clay content and pH to that of Australian desert areas^86^. The global prediction of k_ref_ in this study reflects the spatial distribution characteristics of the predictive factors and is therefore valuable for developing and testing global scale models.

In conclusion, our study based on laboratory incubation data illustrates the controlling role of various edaphic-climatic predictors for the expected decomposition of different SOM pools. Generally, soil texture, particularly clay content, dominates the active SOM pool turnover presumably by limiting water holding capacity as well as oxygen diffusion, whereas pH determines the reference decomposition rate of the passive or stable SOM pool probably by regulating microbial activities of bacteria and fungi. In addition to soil texture and pH, climatic conditions (particularly MAP), SOC content, and terrain slope exhibit secondary influence on the SOM decomposition rates. Because of the non-monotonous impacts and spatial variation of influencing factors, the global prediction of kinetic parameters shows prominent regional characteristics. Notably, limited or even missing data for areas such as the Arctic, the Sahara Desert and the Amazon Rainforest weaken our confidence in predictions for these areas. However, our results exhibit important implications for the generalized application of the first-order kinetics assumption. Applying the results from this study could further improve the parameterization of ESMs at the global scale to yield more robust estimates of land-atmosphere carbon fluxes, advancing our understanding of carbon-climate feedbacks under a changing climate.

## Methods

### First-order kinetics model

The decomposition of soil organic matter (SOM) is catalyzed by various enzymes secreted by microbes or plants, involving a variety of biochemical and physical reactions which have not yet been fully understood. To mathematically describe the SOM decomposition processes, many studies simplify the internal mechanism and follow the first-order kinetics assumption with the differential equation expressed as:^87,88^

1 $$\begin{array}{c}\frac{{dS}}{{dt}}=-k\cdot S\end{array}$$

where *S* is the substrate concentration (mass units, e.g., mg C g^–1^ soil), and *k* is the decomposition rate constant (time units, e.g., d^−1^ or year^−1^) of the substrate.

The analytic solution to Eq. (1) is:

2 $$\begin{array}{c}S\left(t\right)=S\left(0\right)\cdot {e}^{-{kt}}\end{array}$$

where *S*(*t*) is the substrate concentration at time *t*, and *S*(0) is the initial substrate concentration. When it comes to multiple SOM pools, the form of the solution can be expressed as:

3 $$\begin{array}{c}S\left(t\right)=S\left(0\right)\cdot \mathop{\sum }\limits_{i=1}^{n}\left({f}_{i}\cdot {e}^{-{k}_{i}t}\right)\end{array}$$

4 $$\begin{array}{c}\mathop{\sum }\limits_{i=1}^{n}{f}_{i}=1\end{array}$$

where *n* is the number of SOM pools, which is usually set to two or three corresponding to a two-pool or three-pool model; *f*_*i*_ is the initial fraction of SOM pool *i*; and *k*_*i*_ is the reference decomposition rate of SOM pool *i*.

Equation (3) calculates the remaining substrate of all SOM pools at time *t*. Subsequently, the total C loss (i.e., cumulative CO_2_ flux, denoted by R_cum_(t)) and the mineralized C flux rate R(t) could be calculated by the following expressions:

5 $$\begin{array}{c}{R}_{{{{{{\rm{cum}}}}}}}\left(t\right)=S\left(0\right)\cdot \left(1-\mathop{\sum }\limits_{i=1}^{n}{f}_{i}\cdot {e}^{-{k}_{i}t}\right)\end{array}$$

6 $$\begin{array}{c}R\left(t\right)=\mathop{{{{{{\rm{lim}}}}}}}\limits_{\triangle t\to 0}\frac{{R}_{{{{{{\rm{cum}}}}}}}\left(t+\triangle t\right)-{R}_{{{{{{\rm{cum}}}}}}}\left(t\right)}{\triangle t}=S\left(0\right)\cdot \mathop{\sum }\limits_{i=1}^{n}{k}_{i}\cdot {f}_{i}\cdot {e}^{-{k}_{i}t}\end{array}$$

### Incubation dataset compilation

We compiled a global dataset of estimated values of first-order kinetics parameters by fitting against measured data from laboratory incubation experiments conducted pertaining to various climate zones and ecosystems. By setting keywords to first-order, incubation, SOM, soil respiration, multi-pool, two-pool or three-pool, we searched the Web of Science, Google Scholar and China National Knowledge Infrastructure (CNKI, http://www.cnki. net). Finally, we obtained 859 records from 59 publications with detailed information of evaluation criteria (e.g., coefficient of determination (R^2^), Root Mean Square Error (RMSE), Akaike Information Criterion (AIC), and/or Bayesian Information Criterion (BIC)) and fitted first-order kinetics parameters including the reference decomposition rates and the initial pool sizes. We recorded experimental information such as geographic location, elevation (Elev, units: m), mean annual precipitation (MAP, units: mm), mean annual temperature (MAT, units: °C) and ecosystem type of the sampling location, soil texture (sand, silt, and clay fractions, units: %), soil pH, and soil moisture (units: percentages of soil water holding capacity), incubation temperature (IncT, units: °C), incubation experiment duration (units: day), measured variables (CO_2_, CO_2_ + CH_4_, ^13^CO_2_), fitted values of kinetic parameters (SOM pool relative sizes, units: %; reference decomposition rate, units: d^−1^) and model evaluation indexes (i.e., R^2^, RMSE, AIC, and BIC) (Supplementary Data).

In the case that the fitted kinetics parameters were presented in the form of graphs, we extracted the values by using WebPlotDigitizer 4.5 (https://apps.automeris.io/wpd/index.zh_CN.html). For studies not providing the fitted kinetics parameters (relative pool sizes and decay rates), we used a two- or three-pool first-order kinetics model to fit the measured soil respiration data by the Nonlinear Least Squares (NLS) method^89^, where we chose the Gauss-Newton algorithm to minimize the residual sum of squares (RSS). To picture the global distribution of incubation experiments, the study sites were displayed on the map, most of which were distributed between 25 and 50 degrees north latitude especially in North America and China.

To check the reliability of the compiled dataset, we collected the default values of kinetic parameters of well-known models, such as the Community Land Model with Carbon Nitrogen Biogeochemistry (CLMcn)^13^, Daily Century Model (DAYCENT)^48^, Agricultural Nitrogen Model (ANIMO)^46^ and DAISY^47^. We converted all the reference decomposition rates to the same units (i.e., d^–1^).

### Controlling factors identification and global datasets collection

To analyze the impact of edaphic-climate conditions on the SOM decomposition kinetics parameters, we selected numerical factors as independent variables from our compiled dataset, such as MAP, MAT, sand and clay fraction (Sand and Clay), pH, Elev, and IncT. Although we used data from laboratory incubations, we also included MAP and MAT for the soil origin locations to reflect the effect of regional climate characteristics. We involved sand and clay fraction to characterize the effect of soil texture. Simultaneously, we included pH to represent the soil acidity and alkalinity impact. Elevation and laboratory incubation temperature represent the topographic influence and the environmental impact of lab incubation experiments, respectively. Soil moisture in these laboratory incubation experiments was usually maintained at a constant value (i.e., 60% water holding capacity), which is too homogeneous to be treated as an independent variable in this study. Apart from the seven documented variables, we included soil organic carbon (SOC) and microbial biomass carbon (MBC) as the explanatory variables as they directly characterize SOM and microbial community, respectively^90^. In addition to elevation, terrain slope (Slope) was considered for its significant impact on SOM storage by affecting migration and transformation of soil nutrient^71^. We used the normalized difference vegetation index (NDVI), a commonly used indicator of vegetation coverage^91^, as a proxy of vegetation for predicting the SOM decomposition kinetics parameters.

We collected global datasets of the aforementioned eleven explanatory variables to fill missing values of the compiled dataset and predict the global patterns of first-order kinetic parameters. The WorldClim version 2.1 monthly historical climate data, released in January 2020, was chosen to analyze the impact of climatic factors, which is an average of the period 1970−2000 and available at spatial resolutions between 1 to 340 square kilometers^92^. We downloaded and calculated MAP and MAT with a 1-km spatial resolution. For global soil properties data, we referred to the SoilGrids version published in 2017 from the International Soil Reference and Information Centre (ISRIC), including sand, clay, and silt fraction (%), soil organic carbon (g kg^−1^) and pH values at a 1-km spatial resolution^93^. Global MBC data was obtained through the Oak Ridge National Laboratory (ORNL) Distributed Active Archive Center (DAAC)^94^, which was compiled from a comprehensive survey of publications from the late 1970s to 2012. For global elevation data, we integrated the sixteen blocked DEM datasets with 1 km spatial resolution from National Centers for Environmental Information (NCEI) of National Oceanic and Atmospheric Administration (NOAA) (https://www.ngdc.noaa.gov/mgg/topo/DATATILES/elev/) to obtain a global distribution of terrestrial elevation and calculated terrain slope by ArcGIS 10.2. Global NDVI data was obtained through the NASA Making Earth System Data Records for Use in Research Environments (MEaSUREs) Vegetation Index and Phenology (VIP) global datasets (doi:10.5067/MEaSUREs/VIP/VIPPHEN_NDVI.004), containing yearly average of the period 1981−2014 and available at 0.05-degree spatial resolutions.

Notably, the adopted global soil properties (Sand, Clay, pH, and SOC) datasets remove the Antarctic part covered by glaciers and only include 60 degrees south to 90 degrees north latitude, while the other datasets (MAP, MAT, Elev, Slope, MBC, and NDVI) cover all latitudes of the globe. We kept them within the same extent ranging from 60 degrees south and 90 degrees north latitude by applying the function “crop” in R package “raster”.

### Statistical analysis

The non-parametric Kruskal-Wallis (KW) test was adopted to investigate the difference of fast and slow pool kinetic parameters (especially the reference decomposition rates, i.e., k_ref_) between the two-pool and three-pool model at a significance level of 0.05. To verify whether these variables were independent of each other, the Spearman Correlation Analysis (SCA), which does not require the variables to satisfy a normal distribution, was employed to detect the correlation between any two of the eleven explanatory variables^95^. All statistical analysis was carried out using R software 4.0.2^96^. Correlation strength is classified as per Xia (2020), utilizing the Spearman correlation coefficient (*ρ*) values and significance test index p-values.

### Feature selection and predictive modelling

We selected MAP, MAT, Sand, Clay, pH, SOC, MBC, Elev, Slope, NDVI, and IncT as explanatory variables. More parameters usually contribute to better model performance, but also lead to higher model complexity and uncertainty. To obtain the optimal combination of independent variables, we used recursive feature elimination (RFE) method, an effective feature selection method for regression trees models^97^, to screen out unimportant variables. Specifically, we used the function “rfe” in R package “caret” to train the models with different predictors combinations based on 10-fold cross-validation and elected the optimal combination of independent variables by maximizing the goodness-of-fit between predicted and observed SOM decomposition kinetics parameters.

To erect a reliable relationship between kinetic parameters and explanatory predictors, we elected the multivariable linear regression (MLR)^98^, gradient boosting machine (GBM) and random forest (RF)^99^. The MLR, with its simple model structure, is commonly used as a statistical approach to describe the linear association of independent variables with one dependent variable^100^. The GBM, one of the boosting methods, is an efficient machine learning algorithm for dealing with regression and classification problems, where sequential decision trees are trained and linearly integrated to minimize the loss function of the previously trained decision trees on the gradient descent direction^101^. The RF, widely used in many research fields for detecting nonlinear associations, is a powerful machine learning approach that can avoid overfitting by growing each tree of all decision trees^102^. There are four hyperparameters in GBM model, including the number of trees (i.e., n.trees), complexity of the tree (i.e., interaction.depth), learning rate (i.e., shrinkage), and the minimum number of training set samples in a node to commence splitting (i.e., n.minobsinnode)^103^. To optimize the hyperparameters of GBM, we adopted the grid search method^104^ by setting n.trees to 10–200, interaction.depth to 1–7, and shrinkage to 0.01 and 0.1, while keeping n.minobsinnode to a constant value (i.e., 10)^103^. For RF, we set the maximum number of allowed trees to 100 and controlled the only one user-selected parameter mtry, the numbers of covariates used in tree splits, between 2 and the number of independent variables minus 1.

We evaluated modeling performance with metrics such as coefficient of determination (R^2^)^105^, concordance correlation coefficient (*ρ*_*c*_)^51^, RMSE^106^, RMSEn and AIC:^107^

7 $$\begin{array}{c}{R}^{2}=1-\frac{{\sum }_{i=1}^{n}{\left({y}_{{{{{{\rm{sim}}}}}}}^{i}-{y}_{{{{{{\rm{obs}}}}}}}^{i}\right)}^{2}}{{\sum }_{i=1}^{n}{\left({y}_{{{{{{\rm{obs}}}}}}}^{i}-{\bar{y}}_{{{{{{\rm{obs}}}}}}}\right)}^{2}}\end{array}$$

8 $$\begin{array}{c}{\rho }_{c}=\frac{2\cdot {\sum }_{i=1}^{n}\left({y}_{{{{{{\rm{sim}}}}}}}^{i}-{\bar{y}}_{{{{{{\rm{sim}}}}}}}\right)\left({y}_{{{{{{\rm{obs}}}}}}}^{i}-{\bar{y}}_{{{{{{\rm{obs}}}}}}}\right)}{n\cdot {\left({\bar{y}}_{{{{{{\rm{sim}}}}}}}-{\bar{y}}_{{{{{{\rm{obs}}}}}}}\right)}^{2}+{\sum }_{i=1}^{n}\left[{\left({y}_{{{{{{\rm{sim}}}}}}}^{i}-{\bar{y}}_{{{{{{\rm{sim}}}}}}}\right)}^{2}+{\left({y}_{{{{{{\rm{obs}}}}}}}^{i}-{\bar{y}}_{{{{{{\rm{obs}}}}}}}\right)}^{2}\right]}\end{array}$$

9 $$\begin{array}{c}{RMSE}=\sqrt{\frac{{\sum }_{i=1}^{n}{\left({y}_{{{{{{\rm{sim}}}}}}}^{i}-{y}_{{{{{{\rm{obs}}}}}}}^{i}\right)}^{2}}{n}}\end{array}$$

10 $$\begin{array}{c}{RMSEn}=\frac{{RMSE}}{{y}_{{{{{{\rm{obs}}}}}}}^{{{{{{\rm{Q}}}}}}3}-{y}_{{{{{{\rm{obs}}}}}}}^{{{{{{\rm{Q}}}}}}1}}\end{array}$$

11 $$\begin{array}{c}{AIC}=n\cdot {{{{{\rm{ln}}}}}}\frac{{\sum }_{i=1}^{n}{\left({y}_{{{{{{\rm{sim}}}}}}}^{i}-{y}_{{{{{{\rm{obs}}}}}}}^{i}\right)}^{2}}{n}+2\cdot {p }\end{array}$$

where *n* is the number of observations, *p* is the number of explanatory variables; ${y}_{{{\mbox{obs}}}}^{i}$ and ${y}_{{{\mbox{sim}}}}^{i}$ denote the *i*th observed and simulated value, respectively; ${\bar{y}}_{{{\mbox{obs}}}}$ and ${\bar{y}}_{{{\mbox{sim}}}}$ are the mean value of observed and simulated data, respectively;$\,{y}_{{{{{{\rm{obs}}}}}}}^{{{{{{\rm{Q}}}}}}1}$ and ${y}_{{{{{{\rm{obs}}}}}}}^{{{{{{\rm{Q}}}}}}3}$ represent the first and third quartile of the observations, respectively. A higher R^2^ and *ρ*_*c*_, and a lower RMSEn and AIC, represents better model performance.

We developed the predictive model with regard to eight kinetics parameters, i.e, M2-k1_ref_, M2-k2_ref_, M2-f1, M3-k1_ref_, M3-k2_ref_, M3-k3_ref_, M3-f1, and M3-f2, where M2 and M3 denote the two-pool model and the three-pool model, respectively; k1_ref_, k2_ref_, and k3_ref_ are the reference decomposition rates of the fast, slow, and passive SOM pools, respectively; and f1 and f2 denote the relative sizes of the fast and slow SOM pools, respectively. For each kinetics parameter, we constructed MLR, GBM, and RF models based on the compiled dataset with and without feature selection by applying the basic functions of “lm”, “gbm” and “rf” method in the R package “caret”, respectively.

We used the function ‘createDataPartition’ in the R package ‘caret’ to partition the training and testing datasets. That is to say, we trained the models by randomly selecting 75% of the full dataset and tested with the remaining 25% of the dataset. Alternatively, k-fold cross-validation can be used as a model validation method, where the data for model training is further partitioned into k equal subsets and each subset is left out for validation while the remaining subsets are used for model training^101,108^. To reduce the uncertainty of stochastic sampling and find the best predictive models, we trained the machine learning models for 100 times and searched the optimal models by maximizing R^2^. In each run, we used repeated ten-fold cross-validation as the resampling method and set the repeat times to three while model training. We examined the three approaches (MLR, GBM, and RF) with split-sample (i.e., 75% for training and 25% for testing) and full dataset separately. We selected the best predictive model as per the lowest AIC.

### Relative importance and partial dependence analysis of explanatory variables

For each kinetic parameter, we estimated the relative importance of explanatory predictors by applying “varImp” function in the R package “caret” to the best predictive model^109^. The values of relative importance of all variables were summed up to be 100 (%). To reveal how the SOM kinetic parameters respond to the changes in explanatory predictors, we conducted the partial dependence analysis by using function “partial” in the R package “pdp” and normalized the values of all explanatory predictors to 0−1.

### Global prediction of SOM decomposition kinetics parameters

We resampled the global datasets of selected independent variables to a common grid cell (i.e., 1-km spatial resolution) by “bilinear” method and derived global datasets of the SOM decomposition kinetics parameters at a 1-km resolution (about 0.0083°) based on the optimal predictive models. For the analysis of global patterns, we resampled the derived datasets to 0.5° spatial resolution by “bilinear” method in R software 4.0.2 and obtained maps of global prediction of SOM decomposition kinetics parameters.

Notably, the adopted global soil properties (Sand, Clay, pH, and SOC) datasets have predicted values with high uncertainties^97^. However, the uncertainties for these variables at 1 km resolution are not provided publicly except for pH, preventing us from a comprehensive assessment of the uncertainty of our final product caused by these input uncertainties. Therefore, we only quantified the grid-by-rid relative uncertainties of the reference decomposition rates in the three-pool model caused by the uncertainty in pH, whose 90% confidence interval was given. The relative uncertainty (ReUn) of a variable is quantified by

12 $${ReUn}=\frac{{Widt}{h}_{90\%{{{{{\rm{CI}}}}}}}}{{Mean}}$$

where Width_90%CI_ and Mean denote the width of the 90% confidence interval and the mean value, respectively.

Subsequently, the quantification of the ReUn of a predicted variable (e.g., M3-k3_ref_) owing to the pH uncertainty on each grid was similar to Eq. (12), where the Width_90%CI_ was defined as the difference in M3-k3_ref_ predicted by the 95% percentile pH and the 5% percentile pH.

In addition, we derived the grid-by-grid uncertainty due to machine learning model structure by computing the ReUn of a predicted decomposition parameter based on 100 decision trees of the best RF model^99^. This model structure uncertainty was owing to resampling of data and unexplained variability not captured by the current RF model^99^. Subsequently, we calculated the mean values and 90% confidence intervals as per latitudes to analyze the latitudinal patterns of the predicted global kinetic parameters.

### Reporting summary

Further information on research design is available in the Nature Portfolio Reporting Summary linked to this article.

## Supplementary information

Supplementary information

Description of Additional Supplementary Files

Supplementary Software

Reporting Summary

## Data Availability

The soil decomposition kinetics data generated in this study are provided in the Supplementary Information/Source Data file ‘Supplementary Software.rar’.

## Code availability

Source code is provided in the file ‘Supplementary Software.rar’.
