# Supplementary material for: Global patterns and edaphic-climatic controls of soil carbon decomposition kinetics predicted from incubation experiments
Source: Nat Commun. 2023 Apr 15;14:2171. doi: 10.1038/s41467-023-37900-3 (PMC10105724; doi:10.1038/s41467-023-37900-3)
Supplement: Supplementary file 1 — Supplementary information [file 41467_2023_37900_MOESM1_ESM.pdf]

Supplementary Information

Global patterns and edaphic-climatic controls of soil carbon decomposition kinetics predicted from incubation experiments

Daifeng Xiang[1,2], Gangsheng Wang[1,2*], Jing Tian[1,2], Wanyu Li[1,2]

[1]State Key Laboratory of Water Resources and Hydropower Engineering Science, Wuhan University, Wuhan 430072 China

[2]Institute for Water-Carbon Cycles and Carbon Neutrality, School of Water Resources and Hydropower Engineering, Wuhan University, Wuhan 430072 China

[*]**Corresponding author:**

Gangsheng Wang, *E-mail address*: wanggs@whu.edu.cn

Co-authors:

Daifeng Xiang (xiangdf@whu.edu.cn)

Jing Tian (tianjing97@whu.edu.cn)

Wanyu Li (liwanyu@whu.edu.cn)

Supplementary Figures

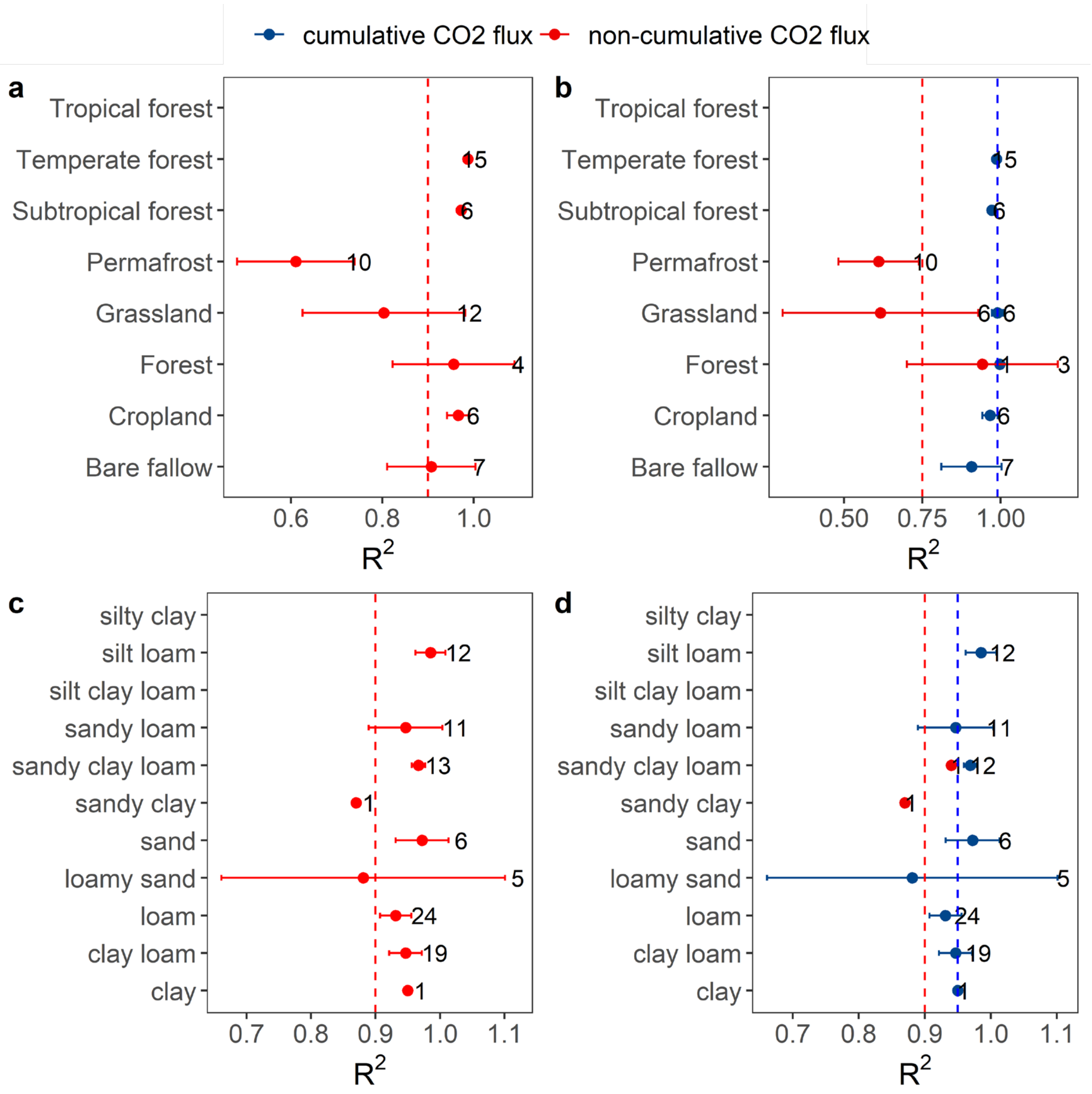

Supplementary Fig. 1. The coefficient of determination (R^2) fitted with experimental data as per ecosystems and soil textures of the compiled dataset. **a**, all data including both cumulative and non-cumulative CO_2 flux data from different ecosystems. **b**, cumulative (blue) and non-cumulative (red) CO_2 flux data from different ecosystems. **c** and **d,** similar to **a** and **b** but for different soil textures. Solid points represent mean values. Error bars delegate 95% confidence intervals. Numbers on the right of error bars denote the number of samples. Dashed lines in **a-d** are equal to 0.9, 0.75 and 0.99, 0.9, 0.9 and 0.95, respectively.

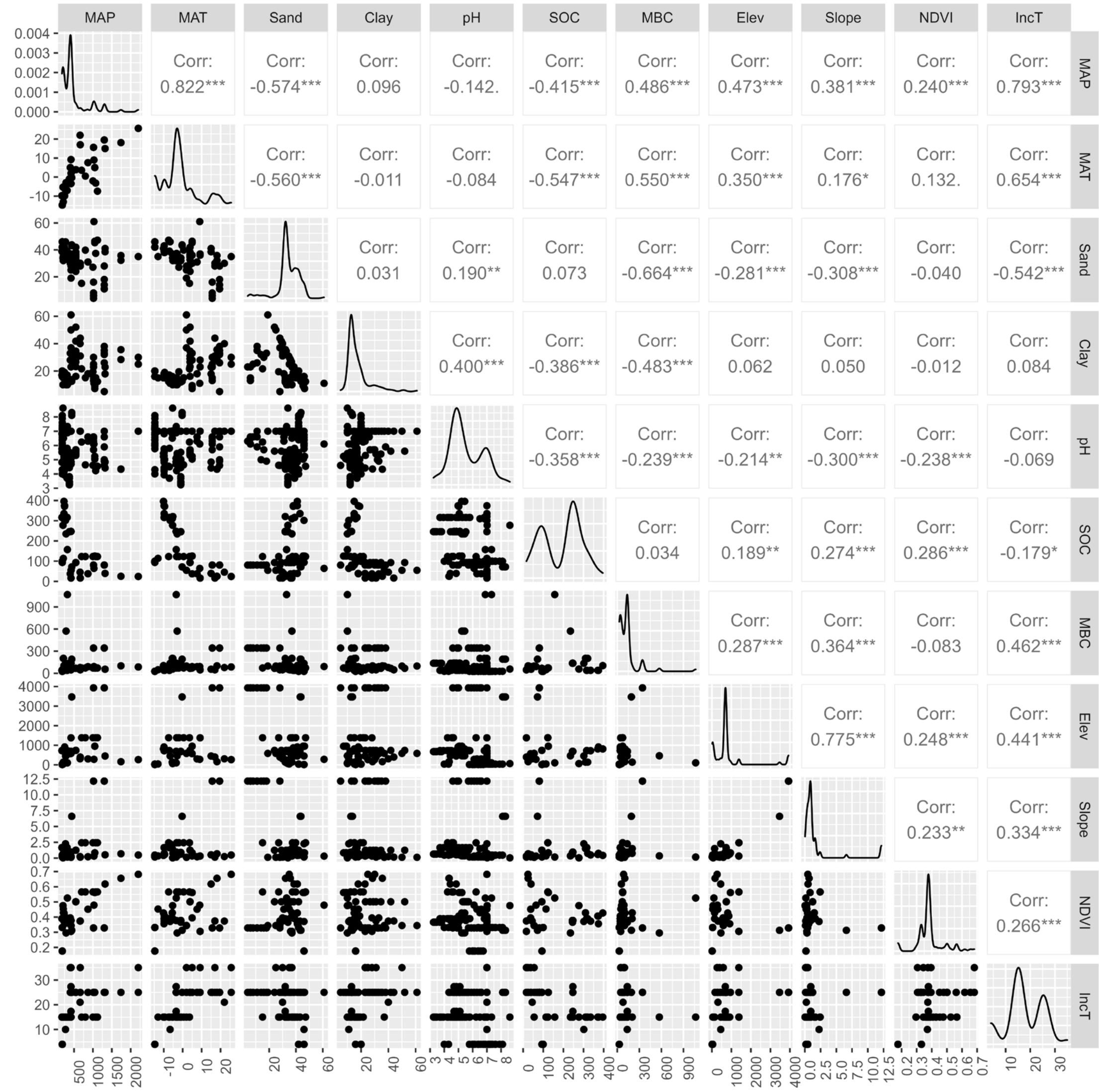

Supplementary Fig. 2. The Spearman correlation between explanatory variables: mean annual precipitation (MAP), mean annual temperature (MAT), sand fraction (Sand), clay fraction (Clay), soil pH, soil organic carbon (SOC), microbial biomass carbon (MBC), elevation (Elev), terrain slope (Slope), normalized difference vegetation index (NDVI), and laboratory incubation temperature (IncT). "***" denotes $p < 0.001$, "**" denotes $p < 0.01$, and "*" denotes $p < 0.05$ as per the one-sided significance test of the Spearman rank correlation coefficient.

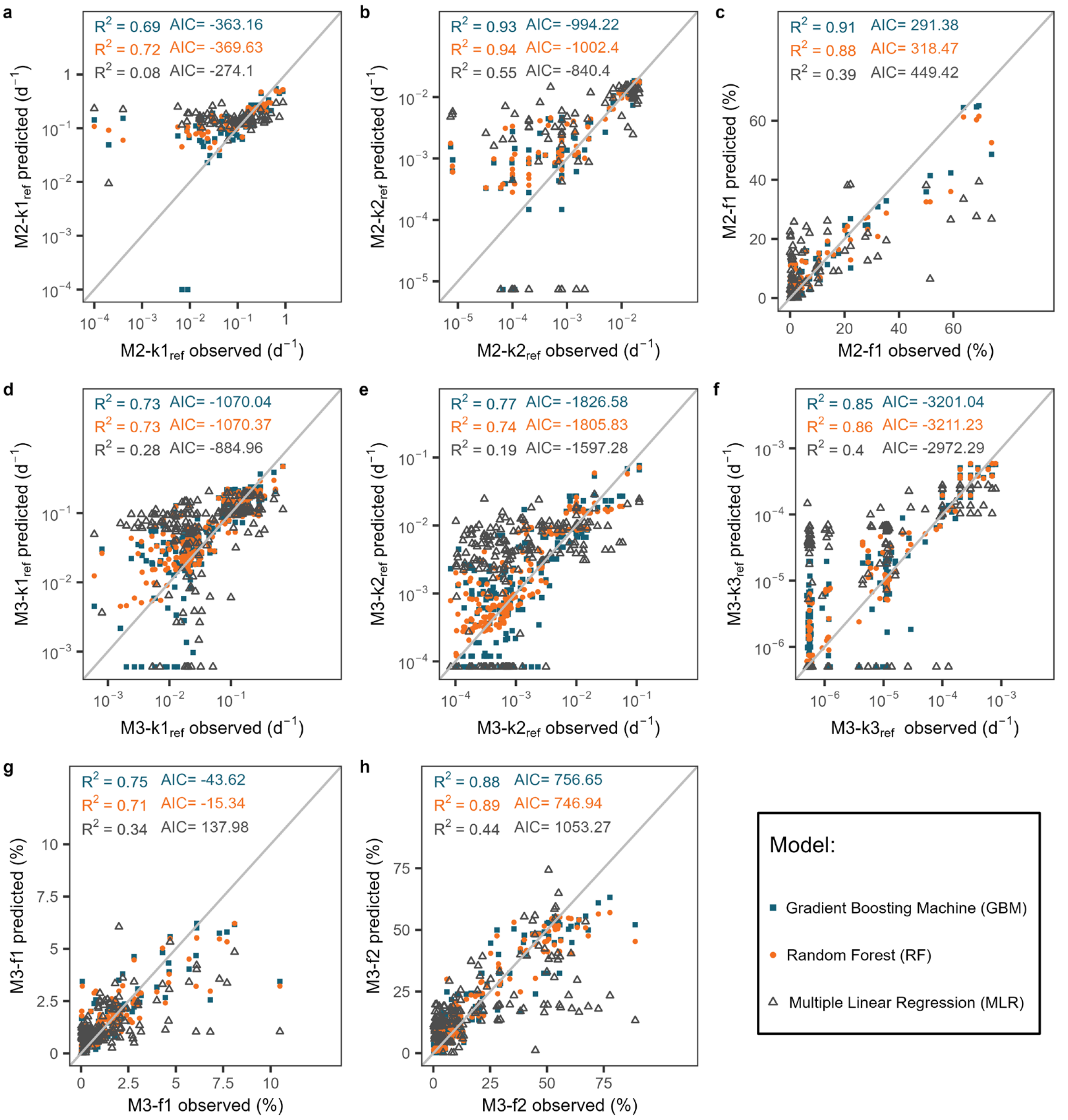

Supplementary Fig. 3. Prediction performance of the Gradient Boosting Machine (GBM), the Random Forest (RF) and the Multiple Linear Regression (MLR) model without feature selection. **a**, **b**, Reference decomposition rate of fast (M2-$k1_{ref}$) and slow pool (M2-$k2_{ref}$) in the two-pool model (M2). **c**, Relative size of the fast pool (M2-f1) in the two-pool model. **d**, **e**, **f**, Reference decomposition rate of fast (M3-$k1_{ref}$), slow (M3-$k2_{ref}$) and passive pool (M3-$k3_{ref}$) in the three-pool model (M3). **g**, **h**, Relative size of the fast (M3-f1) and slow pool (M3-f2) in the three-pool model. R^2 denotes the coefficient of determination. AIC represents the Akaike Information Criterion (see Eq. 11 in Methods).

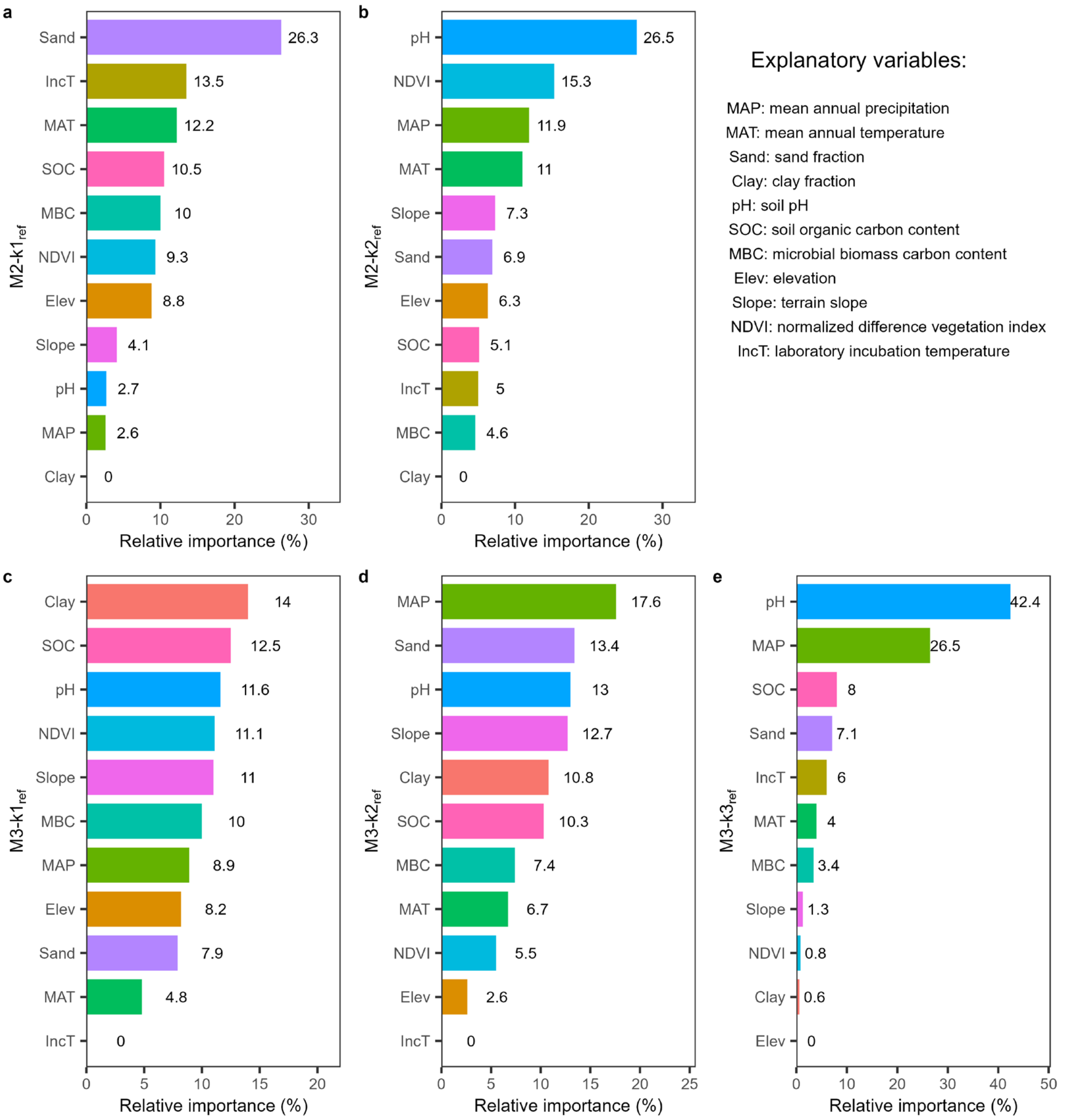

Supplementary Fig. 4. Relative importance of all eleven explanatory variables without feature selection in predicting the reference decomposition rates by the Random Forest (RF) model. **a**, **b**, Variable importance of the fast pool (M2-$k1_{ref}$) and slow pool (M2-$k2_{ref}$) in the two-pool model. **c**, **d**, **e**, Variable importance of fast (M3-$k1_{ref}$), slow (M3-$k2_{ref}$) and passive pool (M3-$k3_{ref}$) in the three-pool model. The sum of the relative importance scores of these variables is 100%.

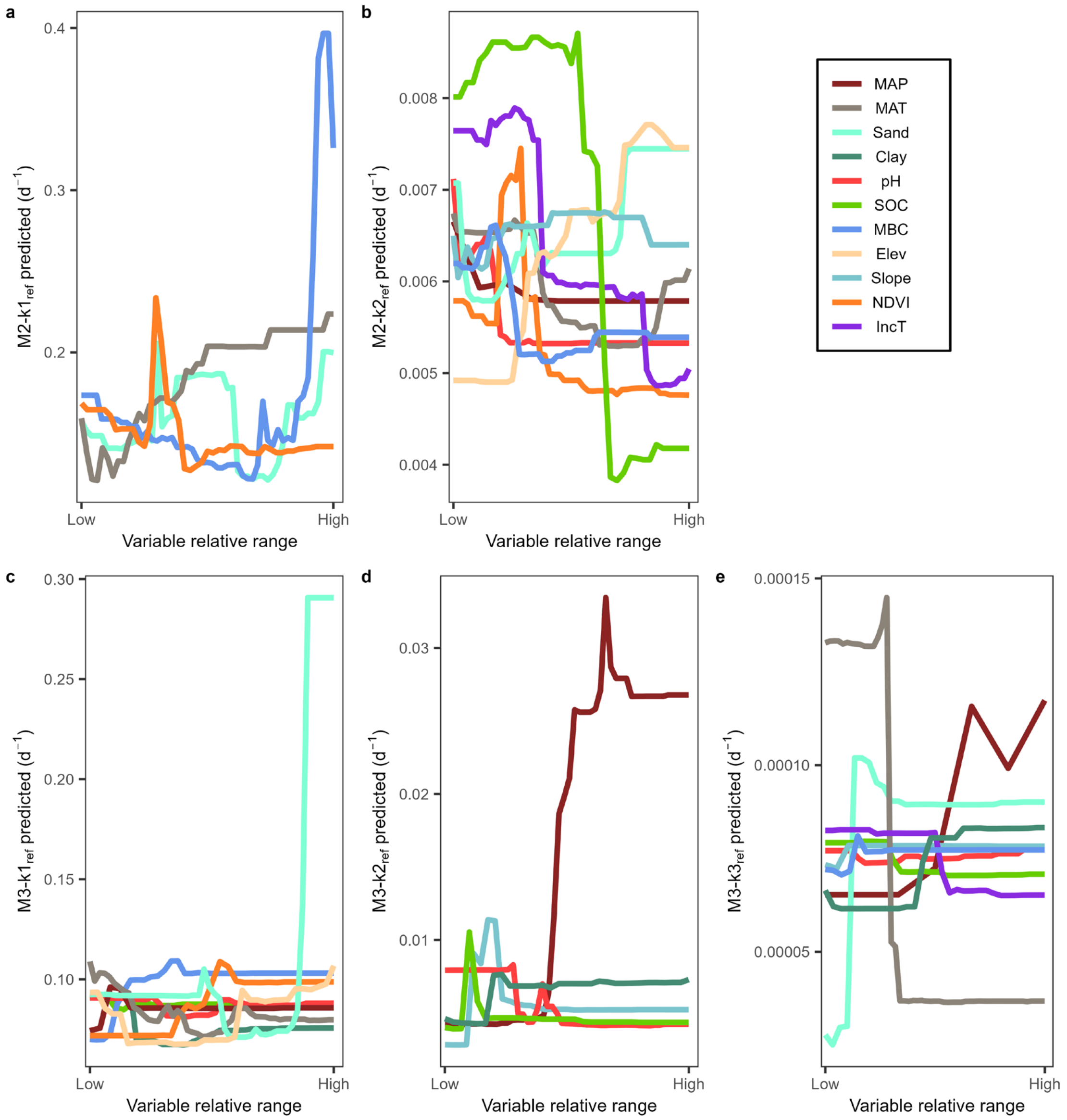

Supplementary Fig. 5. Partial dependence of explanatory variables screened out with feature selection in predicting the reference decomposition rates by the Random Forest (RF) model. **a**, **b**, Partial dependence of the fast pool (M2-$k1_{ref}$) and slow pool (M2-$k2_{ref}$) in the two-pool model. **c**, **d**, **e**, Partial dependence of fast (M3-$k1_{ref}$), slow (M3-$k2_{ref}$) and passive pool (M3-$k3_{ref}$) in the three-pool model.

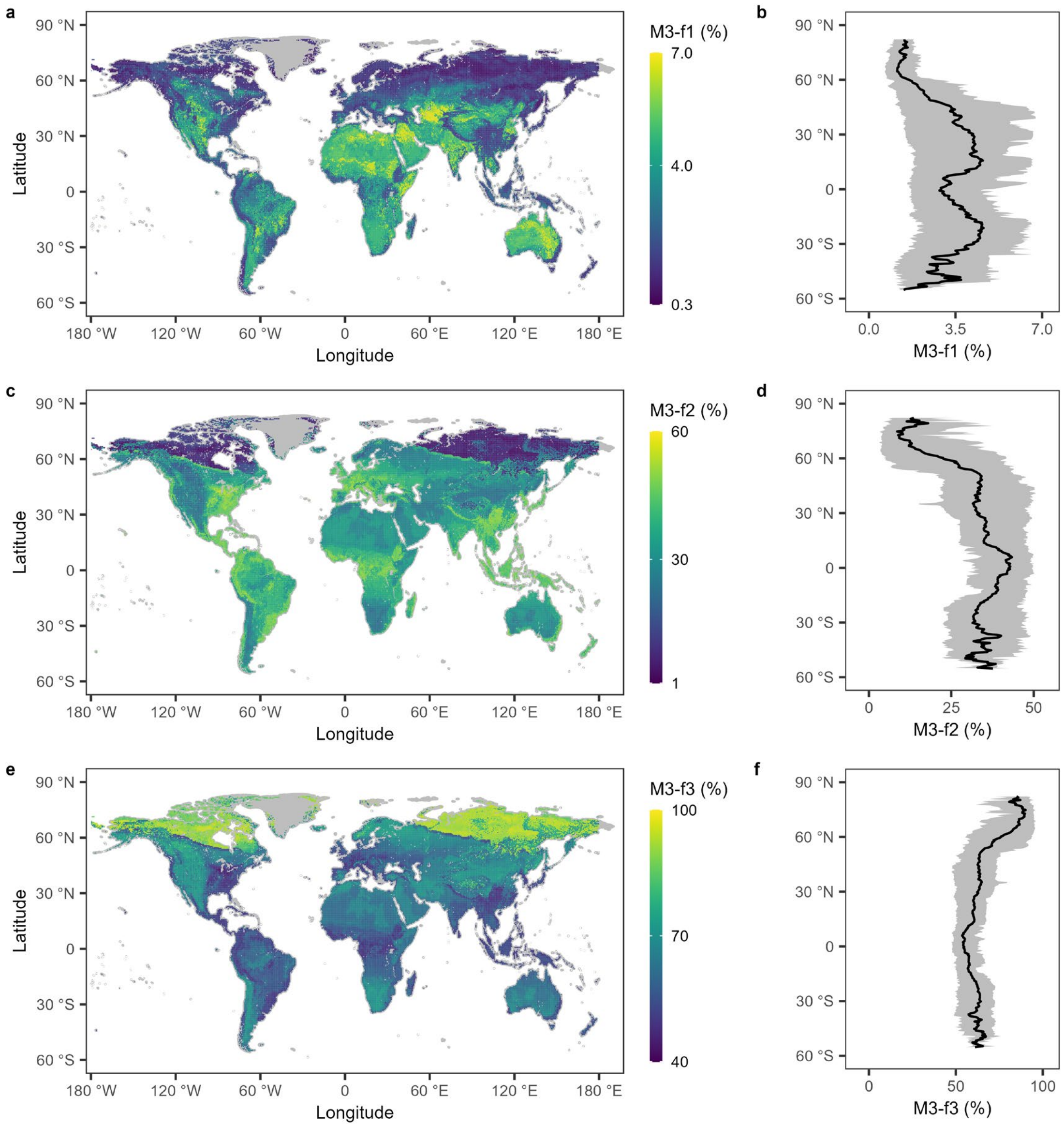

Supplementary Fig. 6. Global distribution and latitudinal pattern of the relative size of the three soil organic matter (SOM) pools predicted by the Random Forest (RF) model with feature selection. **a**, **b**, fast pool (M3-f1). **c**, **d**, slow pool (M3-f2). **e**, **f**, passive pool (M3-f3). Data in **b**, **d** and **f** are presented as mean values and 90% confidence intervals at the respective latitude.

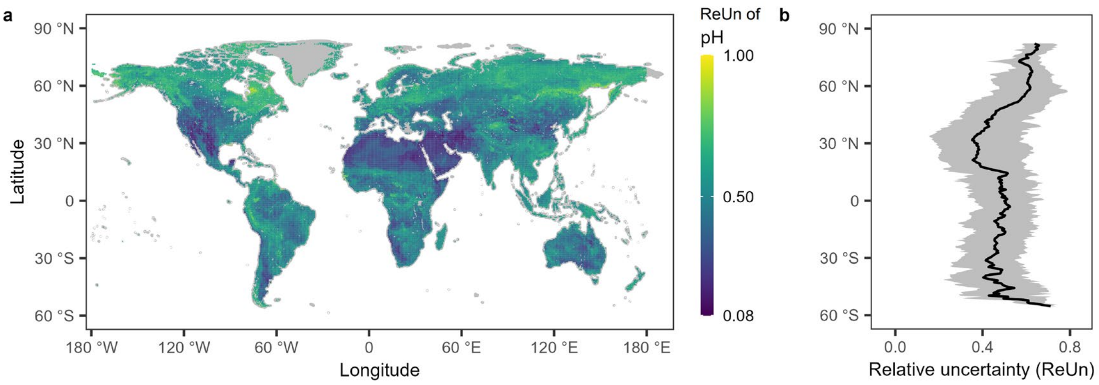

Supplementary Fig. 7. Global relative uncertainty (ReUn) and latitudinal pattern of input data (i.e., soil pH). The uncertainty of pH on each grid in **a** is calculated as $Width_{90\%CI}$/Mean, where $Width_{90\%CI}$ denotes the width of the 90% confidence interval. Data in **b** are presented as mean values and 90% confidence intervals at the respective latitude.

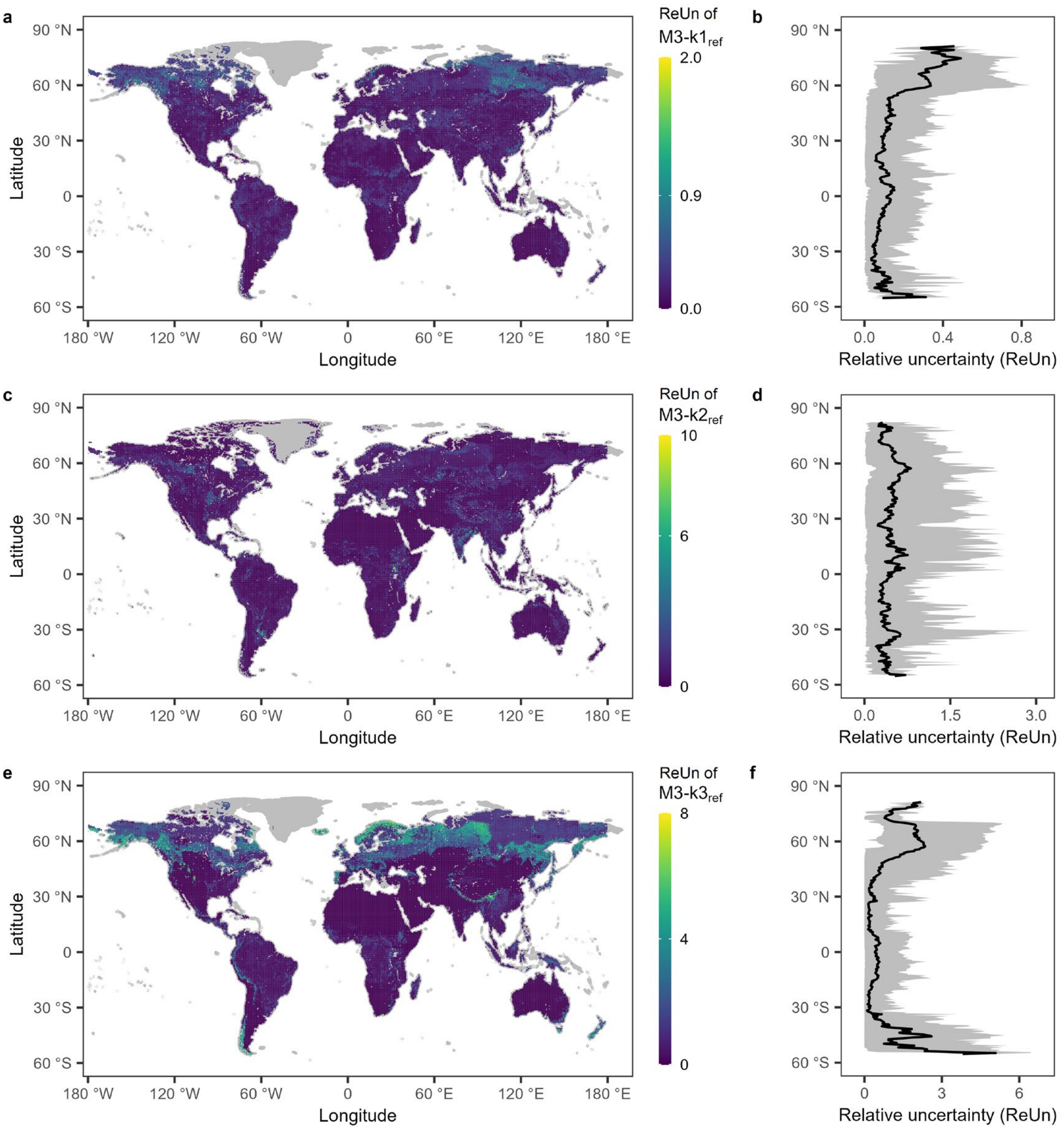

Supplementary Fig. 8. Global relative uncertainty (ReUn) and latitudinal pattern of the reference decomposition rates (k_{ref}) of the three soil organic matter (SOM) pools caused by the uncertainty of input data (i.e., soil pH). **a**, **b**, fast pool (M3-$k1_{ref}$). **c**, **d**, slow pool (M3-$k2_{ref}$). **e**, **f**, passive pool (M3-$k3_{ref}$). For example, we predicted M3-$k3_{ref}$ on a given grid by using the mean, 5% percentile, and 95% percentile of the soil pH value, respectively. The uncertainty of M3-$k3_{ref}$ on this grid was calculated as the ratio of ΔM3-$k3_{ref}$ to M3-$k3_{ref,\ mean}$, where ΔM3-$k3_{ref}$ denotes the difference in M3-$k3_{ref}$ predicted by the 95% percentile pH and the 5% percentile pH, and M3-$k3_{ref,\ mean}$ is the M3-$k3_{ref}$ predicted by the mean pH. Data in **b**, **d** and **f** are presented as mean values and 90% confidence intervals at the respective latitude.

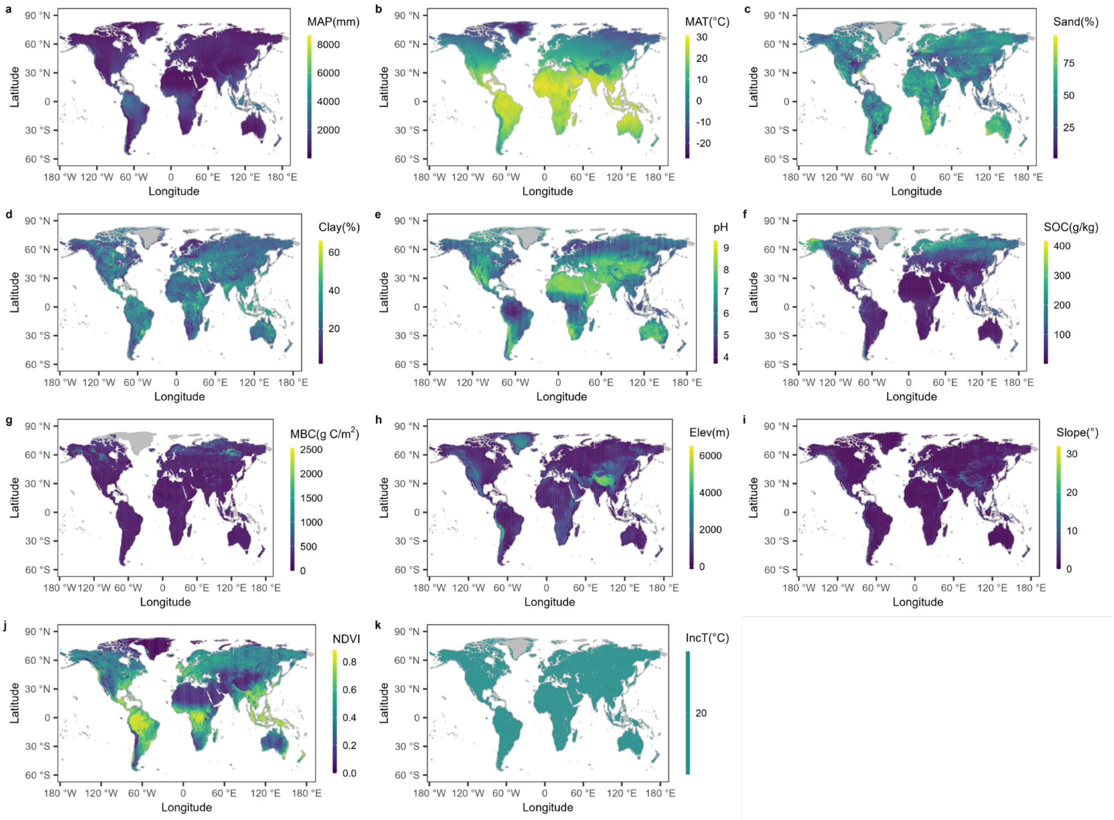

Supplementary Fig. 9. Global distribution of explanatory variables. **a**, Mean annual precipitation (MAP). **b**, Mean annual temperature (MAT). **c**, Sand fraction (Sand). **d**, Clay fraction (Clay). **e**, Laboratory incubation temperature (IncT). **f**, Soil pH in water (pH). **g**, Elevation of the sampling location (Elev) **h**, terrain slope (Slope). **i**, soil organic carbon (SOC). **j**, microbial biomass carbon (MBC). **k**, normalized difference vegetation index (NDVI). The gray color means missing data (**a**-**k**).

Supplementary Table 1. Performance of the Gradient Boosting Machine (GBM), Random Forest (RF) and Multiple Linear Regression (MLR) models trained with and without feature selection. Parentheses indicate negative numbers. M2 and M3 denote the two-pool model and the three-pool model, respectively. $k1_{ref}$, $k2_{ref}$, and $k3_{ref}$ represent the reference decomposition rates of the fast, slow, and passive soil organic matter (SOM) pool, respectively. f1, f2, and f3 refer to the relative sizes (%) of the three SOM pools. R^2 denotes the coefficient of determination (Eq. 7 in Methods). ρ_c denotes concordance correlation coefficient (Eq. 8 in Methods). RMSEn represents the normalized root mean square error (Eq. 9 and 10 in Methods). AIC means Akaike Information Criterion (Eq. 11 in Methods).

		With feature selection				Without feature selection			
		R^2	ρ_c	RMSEn	AIC	R^2	ρ_c	RMSEn	AIC
$M2\text{-}k1_{ref}$	GBM	0.62	0.72	0.61	-359.64	0.69	0.78	0.55	-363.16
	RF	0.7	0.78	0.54	-378.11	0.72	0.79	0.52	-369.63
	RF training	0.66	0.74	0.57	-283.97	0.71	0.78	0.52	-280.07
	RF testing	0.11	0.17	1.07	/	0.04	0.11	1.12	/
	MLR	0.02	0.05	0.97	-283.55	0.08	0.14	0.94	-274.1
$M2\text{-}k2_{ref}$	GBM	0.94	0.97	0.14	-1004.16	0.93	0.96	0.15	-994.22
	RF	0.95	0.97	0.13	-1016.37	0.94	0.97	0.14	-1002.4
	RF training	0.94	0.96	0.15	-757.34	0.93	0.96	0.16	-746.96
	RF testing	0.68	0.81	0.3	/	0.67	0.78	0.39	/
	MLR	0.52	0.67	0.39	-837.41	0.55	0.7	0.38	-840.4
M2-f1	GBM	0.92	0.95	0.5	289.88	0.91	0.95	0.5	291.38
	RF	0.88	0.92	0.59	317.33	0.88	0.92	0.59	318.47
	RF training	0.83	0.88	0.7	265.33	0.83	0.88	0.73	271.45
	RF testing	0.55	0.66	1.02	/	0.49	0.69	1.04	/
	MLR	0.39	0.55	1.33	449.42	0.39	0.55	1.33	449.42
$M3\text{-}k1_{ref}$	GBM	0.7	0.8	0.47	-1051.16	0.73	0.83	0.44	-1070.04
	RF	0.71	0.81	0.46	-1057.52	0.73	0.82	0.44	-1070.37
	RF training	0.66	0.77	0.52	-759.6	0.71	0.81	0.46	-788.17
	RF testing	0.52	0.69	0.5	/	0.39	0.55	0.79	/
	MLR	0.17	0.27	0.77	-861.21	0.28	0.43	0.72	-884.96
$M3\text{-}k2_{ref}$	GBM	0.71	0.81	2.45	-1798.49	0.77	0.85	2.2	-1826.58
	RF	0.75	0.84	2.27	-1826.46	0.74	0.83	2.33	-1805.83
	RF training	0.65	0.76	2.54	-1360.22	0.71	0.8	2.7	-1326.31
	RF testing	0.57	0.67	3.82	/	0.37	0.73	1.6	/
	MLR	0.1	0.16	4.31	-1588.63	0.19	0.29	4.08	-1597.28
$M3\text{-}k3_{ref}$	GBM	0.85	0.92	0.58	-3205.12	0.85	0.92	0.58	-3201.04
	RF	0.86	0.93	0.55	-3219.14	0.86	0.92	0.56	-3211.23
	RF training	0.84	0.91	0.58	-2423.63	0.84	0.91	0.57	-2420.56
	RF testing	0.77	0.83	0.77	/	0.83	0.91	0.68	/
	MLR	0.38	0.52	1.18	-2968.88	0.4	0.55	1.15	-2972.29
M3-f1	GBM	0.61	0.75	0.74	28.39	0.75	0.85	0.59	-43.62

	RF	0.64	0.77	0.71	13.32	0.71	0.81	0.64	-15.34
	RF training	0.62	0.75	0.73	20.97	0.64	0.75	0.74	24.37
	RF testing	0.55	0.68	0.84	/	0.65	0.73	0.7	/
	MLR	0.29	0.45	1	141.3	0.34	0.51	0.96	137.98
M3-f2	GBM	0.87	0.93	0.29	763.6	0.88	0.94	0.28	756.65
	RF	0.88	0.93	0.28	755.74	0.89	0.94	0.27	746.94
	RF training	0.87	0.93	0.32	574.44	0.82	0.89	0.34	637.96
	RF testing	0.54	0.7	0.61	/	0.67	0.79	0.54	/
	MLR	0.32	0.48	0.67	1080.34	0.44	0.61	0.6	1053.27
