# Supplementary material for: Global patterns and edaphic-climatic controls of soil carbon decomposition kinetics predicted from incubation experiments
Source: Nat Commun. 2023 Apr 15;14:2171. doi: 10.1038/s41467-023-37900-3 (PMC10105724; doi:10.1038/s41467-023-37900-3)
Supplement: Supplementary file 2 — Description of Additional Supplementary Files [file 41467_2023_37900_MOESM2_ESM.docx]

File Name: Supplementary Software.rar

Description: The Supplementary Software folder contains three subfolders.

1. The first subfolder named as ‘globalPredictions_halfDegree’ includes the global predictions of SOM decomposition kinetics parameters (i.e., the reference decomposition rate (k_ref_) and the fraction of each pool (f) in the three-pool model) at half degree spatial resolution.
2. The second subfolder named as ‘sourceCode_inputData’ includes the source code (i.e., ‘globalKineticsEstimation.R’), the collected soil decomposition kinetics data (i.e., ‘compiledDataset_SOM-decompositiion.xlsx’), and the Random Forest model trained with split-sample (i.e., 75% for training and 25% for testing).
3. The third subfolder named as ‘sourceData_of_Figures’ includes the raw data of boxplots (i.e., Fig. 1b-e) and scatter plots (i.e., Fig. 2a-h) of the manuscript and Supplementary Information.
