# Supplementary material for: Global patterns and edaphic-climatic controls of soil carbon decomposition kinetics predicted from incubation experiments
Source: Nat Commun. 2023 Apr 15;14:2171. doi: 10.1038/s41467-023-37900-3 (PMC10105724; doi:10.1038/s41467-023-37900-3)
Supplement: Supplementary file 4 — Reporting Summary [file 41467_2023_37900_MOESM4_ESM.pdf]

nature portfolio

Corresponding author(s): Gangsheng Wang

Last updated by author(s): Mar 29, 2023

Reporting Summary

Nature Portfolio wishes to improve the reproducibility of the work that we publish. This form provides structure for consistency and transparency in reporting. For further information on Nature Portfolio policies, see our Editorial Policies and the Editorial Policy Checklist.

Statistics

For all statistical analyses, confirm that the following items are present in the figure legend, table legend, main text, or Methods section.

n/a	Confirmed	
☐	☒	The exact sample size (*n*) for each experimental group/condition, given as a discrete number and unit of measurement
☐	☒	A statement on whether measurements were taken from distinct samples or whether the same sample was measured repeatedly
☐	☒	The statistical test(s) used AND whether they are one- or two-sided<br>*Only common tests should be described solely by name; describe more complex techniques in the Methods section.*
☐	☒	A description of all covariates tested
☐	☒	A description of any assumptions or corrections, such as tests of normality and adjustment for multiple comparisons
☐	☒	A full description of the statistical parameters including central tendency (e.g. means) or other basic estimates (e.g. regression coefficient) AND variation (e.g. standard deviation) or associated estimates of uncertainty (e.g. confidence intervals)
☐	☒	For null hypothesis testing, the test statistic (e.g. *F*, *t*, *r*) with confidence intervals, effect sizes, degrees of freedom and *P* value noted<br>*Give P values as exact values whenever suitable.*
☐	☒	For Bayesian analysis, information on the choice of priors and Markov chain Monte Carlo settings
☐	☒	For hierarchical and complex designs, identification of the appropriate level for tests and full reporting of outcomes
☐	☒	Estimates of effect sizes (e.g. Cohen's *d*, Pearson's *r*), indicating how they were calculated

Our web collection on statistics for biologists contains articles on many of the points above.

Software and code

Policy information about availability of computer code

Data collection	The data for model training were derived from publications listed as a part of supplementary information. The raw data of global mean annual temperature and precipitation data were available in Fick et al. (2017; see Reference), in WorldClim version 2.1 (https://doi.org/10.1002/joc.5086). The global dataset of soil properties were collected from ISRIC SoilGrids version published in Hengl et al. (2017, https://doi.org/10.1371/journal.pone.0105992). Global DEM data was integrated from NCEI NOAA (https://www.ngdc.noaa.gov/mgg/topo/DATATILES/elev/). Global MBC data was obtained through the Oak Ridge National Laboratory (ORNL) Distributed Active ARchive Center (DAAC). Global NDVI data was obtained through the NASA Making Earth System Data Records for Use in Research Environment (MEaSUREs) Vegetation Index and Phenology (VIP) global datasets (doi:10.5067/MEaSUREs/VIP/VIPPHEN_NDVI.004).
Data analysis	All statistical analysis was carried out using R software 4.0.2. The code of data analysis is provided with this paper.

For manuscripts utilizing custom algorithms or software that are central to the research but not yet described in published literature, software must be made available to editors and reviewers. We strongly encourage code deposition in a community repository (e.g. GitHub). See the Nature Portfolio guidelines for submitting code & software for further information.

Data

Policy information about availability of data

All manuscripts must include a data availability statement. This statement should provide the following information, where applicable:

- Accession codes, unique identifiers, or web links for publicly available datasets
- A description of any restrictions on data availability
- For clinical datasets or third party data, please ensure that the statement adheres to our policy

The model code and data are provided with this paper.

Human research participants

Policy information about studies involving human research participants and Sex and Gender in Research.

Reporting on sex and gender	N/A
Population characteristics	N/A
Recruitment	N/A
Ethics oversight	N/A

Note that full information on the approval of the study protocol must also be provided in the manuscript.

Field-specific reporting

Please select the one below that is the best fit for your research. If you are not sure, read the appropriate sections before making your selection.

☐ Life sciences ☐ Behavioural & social sciences ☒ Ecological, evolutionary & environmental sciences

For a reference copy of the document with all sections, see nature.com/documents/nr-reporting-summary-flat.pdf

Ecological, evolutionary & environmental sciences study design

All studies must disclose on these points even when the disclosure is negative.

Study description	We employed machine-learning techniques and a comprehensive set of climatic, topographic, soil, and SOM decomposition kinetics data to develop models capable of making prediction of the sizes and reference decomposition rates of multiple SOM pools (fast, slow and passive. We also provide a 1-km resolution of global-scale dataset characterizing the spatial variability of the sizes and reference decomposition rates of these SOM pools ,which may improve global biogeochemical model parameterization and predictions.
Research sample	We compiled a global dataset of estimated values of first-order kinetics parameters by fitting against measured data from laboratory incubation experiments conducted pertaining to various climate zones and ecosystems. By setting keywords to first-order, incubation, SOM, soil respiration, multi-pool, two-pool or three-pool, we searched the Web of Science, Google Scholar and China National Knowledge Infrastructure (CNKI, http://www.cnki. net).
Sampling strategy	We obtained 859 records from 59 publications with detailed information of evaluation criteria (e.g., coefficient of determination (R2), Root Mean Square Error (RMSE), Akaike Information Criterion (AIC), and/or Bayesian Information Criterion (BIC)) and fitted first-order kinetics parameters including the reference decomposition rates and the initial pool sizes.
Data collection	We extracted SOM decomposition kinetics data and the corresponding edaphic-climatic predictors pertaining different ecosystems and soil texture at the global scale from publications listed as a part of supplementary information. As for global datasets, we searched for relevant articles (Fick et al. 2017, doi: 10.1002/joc.5086; Hengl et al. 2017, doi: 10.1371/journal.pone.0105992) and websites (https://www.worldclim.org/data/worldclim21.html; https://data.isric.org/; https://www.ngdc.noaa.gov/mgg/topo/DATATILES/elev/; doi:10.5067/MEaSUREs/VIP/VIPPHEN_NDVI.004). All data was collected and downloaded via Microsoft Edge 105.0.1343.33.
Timing and spatial scale	The global mean annual temperature and precipitation data are at 1km spatial resolution, which is an average of the period 1970-2000. The global soil properties data (i.e., sand, clay, and silt fraction, soil organic carbon and pH value) are at 1-km spatial resolution. the global MBC data is at 0.5 degree spatial resolution, which was compiled from a comprehensive survey of publications from the late 1970s to 2012. The global elevation data is at 1-km spatial resolution. The global NDVI data is an average of the period 1981-2014 and available at 0.05-degree spatial resolutions.
Data exclusions	For model training, the records without SOM decomposition kinetics or edaphic-climatic predictors were excluded from the compiled dataset.

Reproducibility	This study is not based on experiments.
Randomization	We trained machine-learning model by randomly select 75% of the full dataset and tested with the remaining 25% dataset. During the model training, we used repeated ten-fold cross-validation as the resampling method.
Blinding	Blinding was not necessary as we did not perform any experiment containing groups and/or treatments.

Did the study involve field work? ☐ Yes ☒ No

Reporting for specific materials, systems and methods

We require information from authors about some types of materials, experimental systems and methods used in many studies. Here, indicate whether each material, system or method listed is relevant to your study. If you are not sure if a list item applies to your research, read the appropriate section before selecting a response.

Materials & experimental systems

n/a	Involved in the study	
☒	☐	Antibodies
☒	☐	Eukaryotic cell lines
☒	☐	Palaeontology and archaeology
☒	☐	Animals and other organisms
☒	☐	Clinical data
☒	☐	Dual use research of concern

Methods

n/a	Involved in the study	
☒	☐	ChIP-seq
☒	☐	Flow cytometry
☒	☐	MRI-based neuroimaging
